# Comparative proteome analysis of embryo and endosperm reveals central differential expression proteins involved in wheat seed germination

**DOI:** 10.1186/s12870-015-0471-z

**Published:** 2015-04-08

**Authors:** Miao He, Chong Zhu, Kun Dong, Ting Zhang, Zhiwei Cheng, Jiarui Li, Yueming Yan

**Affiliations:** College of Life Science, Capital Normal University, Beijing, 100048 China; Department of Plant Pathology, Kansas State University, Manhattan, KS 66506, USA; Hubei Collaborative Innovation Center for Grain Industry, 434025 Jingzhou, China

**Keywords:** Wheat seeds, Germination, Embryo, Endosperm, Proteome, qRT-PCR

## Abstract

**Background:**

Wheat seeds provide a staple food and an important protein source for the world’s population. Seed germination is vital to wheat growth and development and directly affects grain yield and quality. In this study, we performed the first comparative proteomic analysis of wheat embryo and endosperm during seed germination.

**Results:**

The proteomic changes in embryo and endosperm during the four different seed germination stages of elite Chinese bread wheat cultivar Zhengmai 9023 were first investigated. In total, 74 and 34 differentially expressed protein (DEP) spots representing 63 and 26 unique proteins were identified in embryo and endosperm, respectively. Eight common DEP were present in both tissues, and 55 and 18 DEP were specific to embryo and endosperm, respectively. These identified DEP spots could be sorted into 13 functional groups, in which the main group was involved in different metabolism pathways, particularly in the reserves necessary for mobilization in preparation for seed germination. The DEPs from the embryo were mainly related to carbohydrate metabolism, proteometabolism, amino acid metabolism, nucleic acid metabolism, and stress-related proteins, whereas those from the endosperm were mainly involved in protein storage, carbohydrate metabolism, inhibitors, stress response, and protein synthesis. During seed germination, both embryo and endosperm had a basic pattern of oxygen consumption, so the proteins related to respiration and energy metabolism were up-regulated or down-regulated along with respiration of wheat seeds. When germination was complete, most storage proteins from the endosperm began to be mobilized, but only a small amount was degraded during germination. Transcription expression of six representative DEP genes at the mRNA level was consistent with their protein expression changes.

**Conclusion:**

Wheat seed germination is a complex process with imbibition, stirring, and germination stages, which involve a series of physiological, morphological, and proteomic changes. The first process is a rapid water uptake, in which the seed coat becomes softer and the physical state of storage materials change gradually. Then the germinated seed enters the second process (a plateau phase) and the third process (the embryonic axes elongation). Seed embryo and endosperm display distinct differentially expressed proteins, and their synergistic expression mechanisms provide a basis for the normal germination of wheat seeds.

**Electronic supplementary material:**

The online version of this article (doi:10.1186/s12870-015-0471-z) contains supplementary material, which is available to authorized users.

## Background

Wheat (*Triticum aestivum* L.) is one of the three most important grain crops and is widely cultivated worldwide due to its value as a staple food and protein source. Total wheat production is more than 600 million tons, which accounts for more than 20% of the world’s food supply [1]. Wheat grains are primarily composed of the embryo and the endosperm, both of which play important roles in seed germination and subsequent plant growth and development. The embryo forms a radicel, a plumule, and the new plant, whereas the endosperm contains reserve substances to supply nutriments for subsequent plant growth, the basis of wheat yield and quality.

The embryo and endosperm play different roles in wheat seed germination. The embryo contains most of the genetic information that controls germination. Upon imbibition, the substrate and energy starvation activate the embryo to produce phytohormones (mainly gibberellic acid, GA). The GAs can diffuse to aleurone and initiate a signaling cascade that leads to synthesis of α-amylases and other hydrolytic enzymes. These enzymes then secrete into the endosperm to drive the degradation of storage compounds, including starch, lipid, and protein, for seedling establishment [2,3]. Studies of endosperm function have demonstrated that the endosperm can secrete signals to control embryo growth [4]; thus, germination is a systemic response that involves bidirectional interactions between the embryo and endosperm.

Seed germination generally proceeds from heterotrophic to autotrophic and involves a series of physiological, biochemical, and morphological changes. It is highly related to seedling survival rate and subsequent vegetative growth and therefore directly affects wheat yield and quality. Storage materials of starch and protein are mainly deposited in the endosperm. Dry seeds’ water content is usually low (5-15%), and their metabolic activity almost ceases. When the dry seeds imbibe water, germination begins, and when the radicle breaks through the episperm, germination is finished [5]. In general, the seed germination process can be divided into three phases: fast water uptake (phase I), metabolism reactivation (phase II), and radicle emergence (phase III) [6]. Phase II is the most critical stage, because all necessary metabolic pathways and physiological processes are reactivated and germination is initiated in this phase. Germination involves many events, such as proteolysis, synthesis of macromolecules, respiration, changes in subcellular structures, and cell elongation [6,7].

The metabolic pathway of wheat germination is highly complex. Early work reported the activity of some key enzymes in glycolysis, pentose phosphate pathway (PPP), the tricarboxylicacid cycle (TCA cycle), and amino acid metabolism during germination [8]. Aoki et al. (2006) detailed the pathway of starch hydrolysis and sucrose transport during germination [9]. In the embryo, some germination-specific gene products were found, including catabolic enzymes associated with the mobilization of reserves from endosperm [10] and germin (oxalate oxidase), which is related to cell wall restructuring [11]. Caliskan et al. (2003) identified four new members of the wheat “germin” gene family [12]. In the past several decades, most of our knowledge about initiation of germination has come from the *Arabidopsis thaliana* model system [13-15]. Several large-scale -omics methods, including transcriptomic, proteomic, and metabolomic methods, recently have been established to investigate the mechanisms of seed germination [13]. The great achievements in genomics have led to application of large-scale gene expression analysis at both mRNA and protein levels to uncover the features of seed germination. For instance, 51,411 transcripts were identified during the five seed germination stages of elite Chinese bread wheat cultivar Jimai 20 using the Affymetrix Wheat Genome Array [16]. Microarrays containing 2600 genes expressed in developing *Arabidopsis* seeds were reported [17]. At the same time, considerable proteomics work on seed germination in different plants has been performed, such as cress [18], sugar beet [19], *Medicago truncatula* [20], barley [21], maize [22], and rice [23,24]. The development of proteomic techniques has provided a unique opportunity to study the proteome changes during wheat seed germination [25-27].

Although different proteomic studies on seed germination in some plants have been conducted, these investigations were focused only on a single organ or tissue such as whole seeds, endosperm, or embryo. This approach does not provide overall understanding of the metabolic and regulatory networks occurring in both embryo and endosperm tissues. In the present study, we performed the first integrative proteome analysis of embryo and endosperm during wheat seed germination by a comparative proteomic approach. Our results revealed that distinct differentially expressed proteins in seed embryo and endosperm are cooperatively involved in seed germination, which provides new insights into the proteomic mechanisms of wheat seed germination.

## Results

### Seed morphological and structural changes during germination

**Figure 1:**
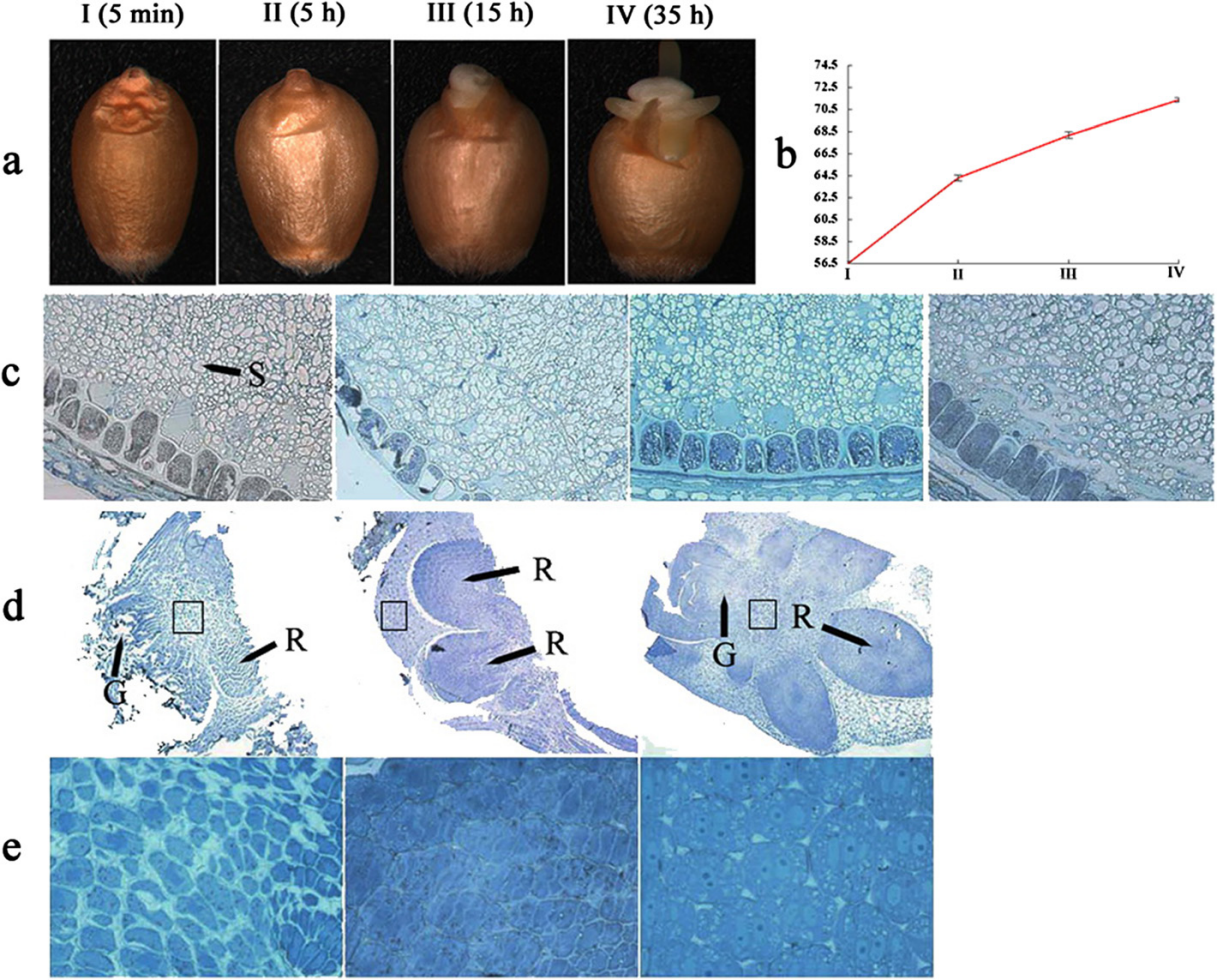
**Morphological and structural characteristics of wheat seeds during the four germination stages. a**. Seed morphological changes during the four germination stages (phase I: seeds absorb water for 5 min; phase II: seeds absorb water for 5 hours; phase III: seeds absorb water for 15 hours; phase IV: seeds absorb water for 35 hours). **b**. Changes in 1,000-seed weight during the germination process. **c**. Endosperm slice from the four germination periods observed by light microscope magnified 20 times (S: starch granules). **d**. Embryo slice from the first three periods observed by light microscope magnified 5 times (R: radicle; G: germ). **e**. Embryo slice from the first three periods observed by light microscope magnified 40 times.

Seed morphological and structural characteristics in the four germination periods are displayed in Figure 1, including phase I (5 min), phase II (5 hours), phase III (15 hours), and phase IV (35 hours). Along with water imbibitions, seed size increased gradually during the seed germination process. As shown in Figure 1a, at phase III, radicels broke through the episperm, indicating that seeds would soon sprout. At phase IV, green germs and three radicles were visible. During the germination process, seed weight increased with imbibition (Figure 1b). In early germination, water uptake is rapid, and after 5 hours, it increased slowly. Endosperm slices from four germination periods were observed by light microscope (Figure 1c), and results showed the endosperm cell structure had changed little from germination stages I to III, but stage IV brought significant change: starch granule sizes were enlarged and starch granule numbers clearly decreased in some areas due to the mobilization and degradation of reserved starch. Observation of embryo slices by light microscope showed that embryo structures displayed significant changes during the germination process (Figure 1d) and gradually disappeared; at phase IV in particular, embryo structures could not be observed due to the emergence of germ and radicles. Results from light microscope observation showed that the embryo cells in the dry seed (phase I) were hydropenic, and thus a larger cell interval was present. Cells expanded when they absorbed water (phase II-III), and cell interval became significantly smaller (Figure 1e).

### Protein expression profiles of embryo and endosperm during seed germination

**Figure 2:**
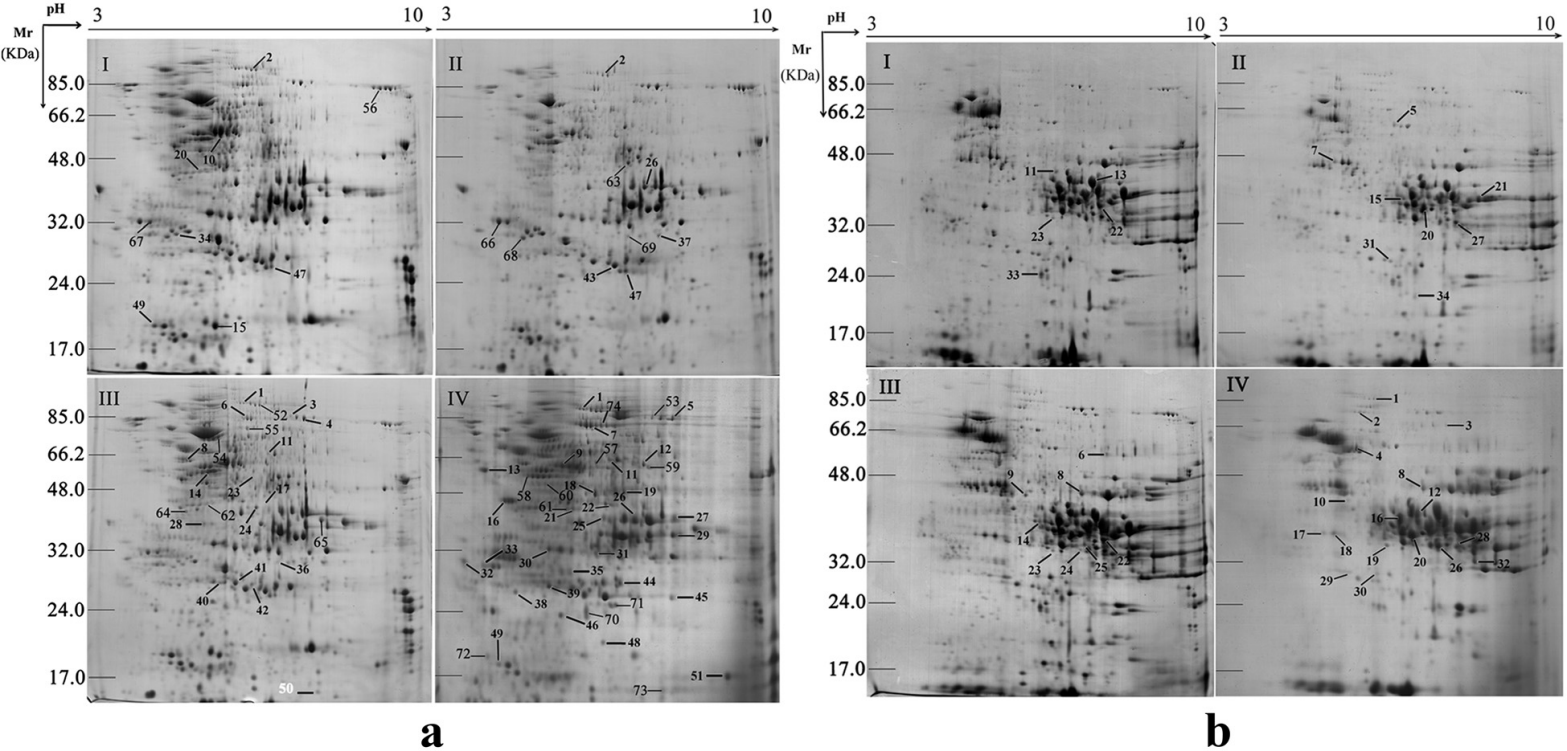
**Proteome maps of wheat embryo (a) and endosperm (b) during four germination periods by linear 2-DE.** The DEP spots with at least 2-fold changes during seed germination are indicated on 2-DE gels.

The embryo and endosperm proteins from four germination periods were separated by two-dimensional electrophoresis (2-DE), and both tissues displayed distinct protein expression profiles on the gel during seed germination (Figure 2). The molecular weight (*M*r) of protein spots from embryos in phase I, II, and III ranged from 15 to 98 kDa, and most were present in pH 4-10. In the last phase, the protein *M*r ranged from 16 to 85 kDa, and most were present in pH 4-7. The protein spots were roughly evenly distributed on the gels (Figure 2a). The protein *M*r from endosperm ranged from 15 to 85 kDa, and most were present in pH 4-10. The protein spots mainly concentrated in a particular area on gels and showed an uneven distribution compared with those in embryo (Figure 2b).

The protein spots on 2-DE gels from four germination periods of embryo and endosperm were compared, and DEP spots with at least two-fold differences in abundance were determined. After image analysis, 97 and 49 DEP spots from embryo (M) and endosperm (N), respectively, were recognized and selected for MALDI-TOF/TOF-MS analysis. In total, 74 and 34 DEP spots representing 63 and 26 unique DEPs from embryo and endosperm were identified, respectively. Eight common DEPs were present in both tissues, and 66 and 26 DEPs were specific to embryo and endosperm, respectively (Additional file 1: Figure S1). Detailed is in Additional file 2: Table S1, and peptide sequences are shown in Additional file 3: Table S3.

**Figure 3:**
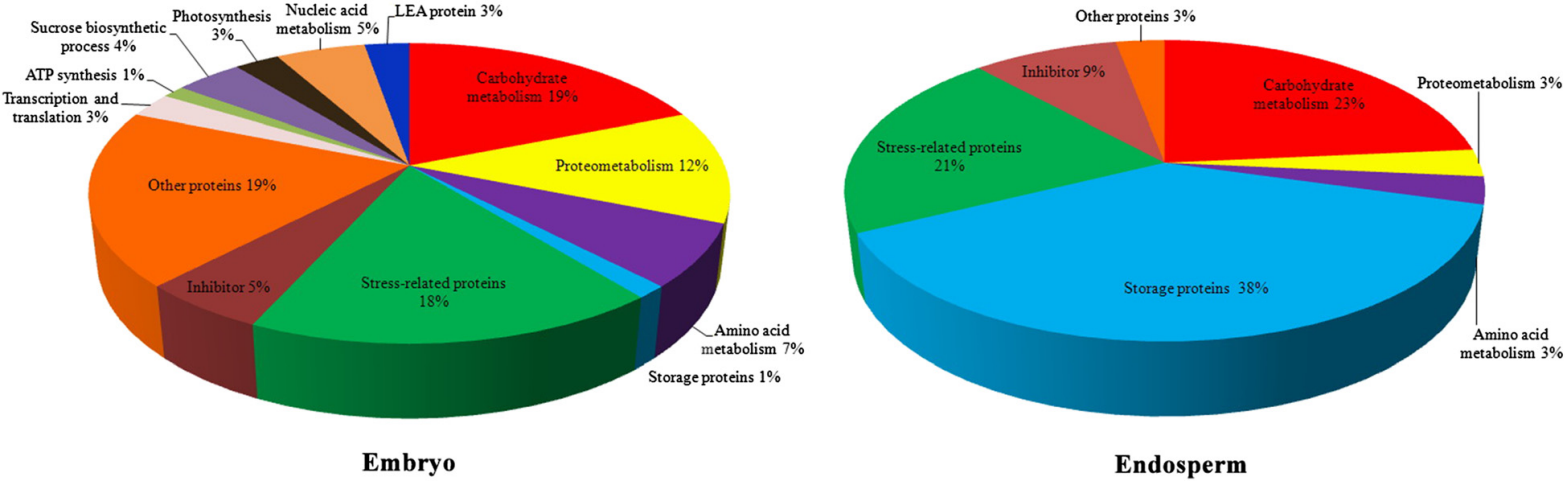
**Functional classification and the proportion of DEP spots identified in wheat embryo and endosperm.**

The identified DEP spots from the embryos were grouped into 13 different functional categories, mainly including stress-induced proteins (18%), carbohydrate metabolism (19%), proteometabolism (12%), nucleic acid metabolism (5%), inhibitors (5%), sucrose biosynthetic process (4%), and other metabolism processes (19%), as shown in Figure 3. The DEPs from endosperm were classified into seven functional categories: storage proteins (38%), carbohydrate metabolism (23%), stress-related proteins (21%), inhibitors (9%), proteometabolism (3%), amino acid metabolism (3%), and other metabolisms (3%) (Figure 3). In order to obtain more information about the predicted proteins, their protein sequences were used as queries to search for homologues by the BLASTP (http://www.ncbi.nlm.nih.gov/BLAST/) tool. The identified corresponding homologues classified into different functional groups are listed in Additional file 2: Table S2. The majority of spots shared above 90% Positive %, Identity %, or Query Coverage % with homologues at the amino acid level, suggesting similar functions. Following this process 15 DEPs remained as having unpredicted functions, namely other proteins metabolism in Additional file 2: Table S1.

**Figure 4:**
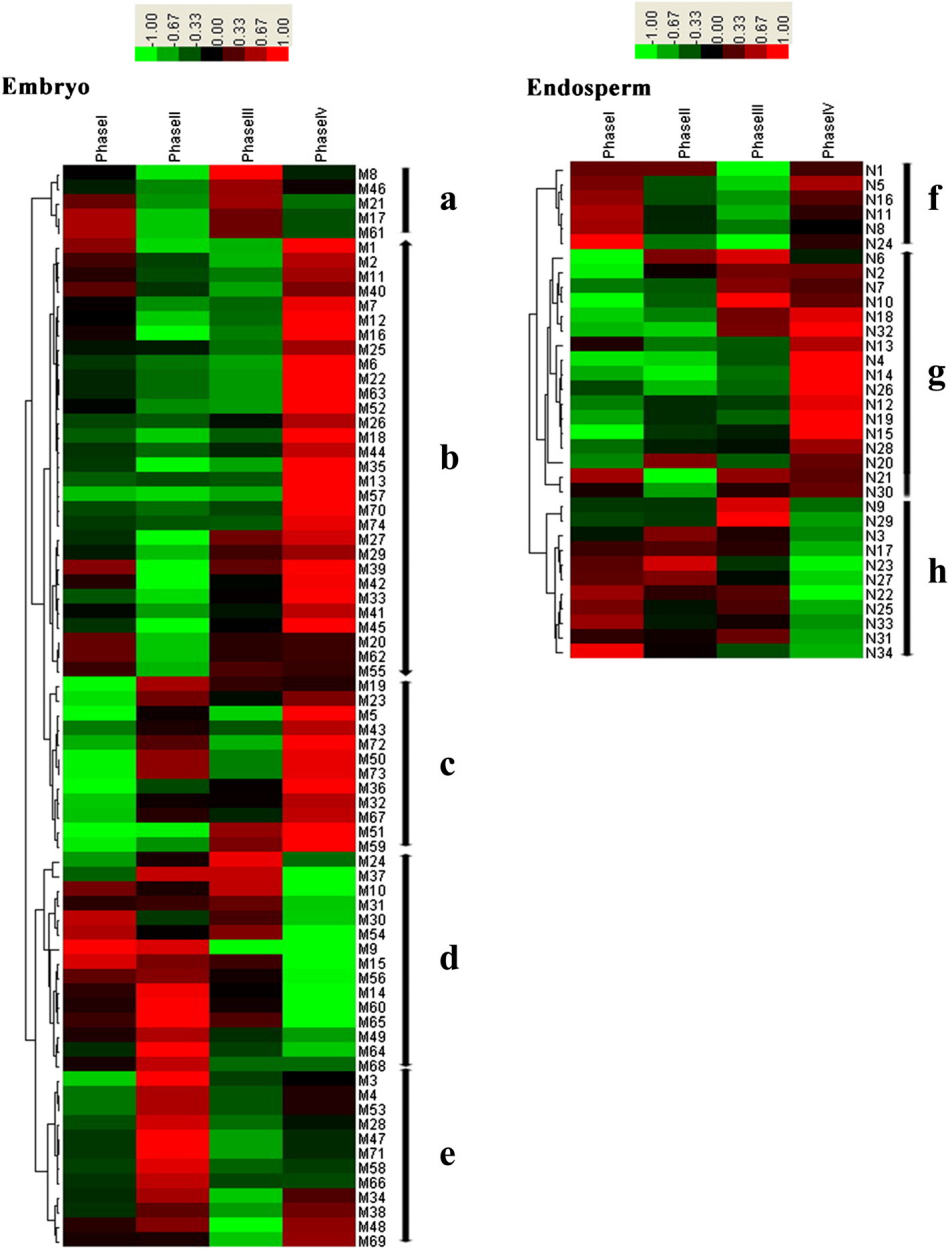
**Hierarchical clustering analysis of the expression patterns of DEP spots in wheat embryo and endosperm during seed germination.** Clustering was based on the protein expression levels across phase I, II, III, and IV of seed germination. Red color indicates a positive abundance in protein spots; green indicates a negative abundance in protein spots. Different expression trends of DEP spots identified in embryo and endosperm were classified. Embryo: **a**. down-up regulation; **b**. up-regulation; **c**. up-down-up regulation; **d**. down regulation; **e**. up-down regulation. Endosperm: **f**. down-up regulation; **g**. up-regulation; **h**. down-regulation.

The transformed Log (protein relative abundance) was used for cluster analysis. The expression pattern analysis of DEPs was performed using the Euclidean distance method over a complete linkage dissimilarity matrix (Figure 4), which can represent quantitative differences in DEPs during the four germination periods. As shown in Figure 4, the embryo had five distinct expression patterns at protein level, designated as A-E. Pattern A displayed a down- to up-regulated expression trend represented by 5 protein spots (5 unique DEPs). Pattern B, the largest group of identified DEPs, had 30 protein spots representing 23 DEPs and showed an up-regulated expression, with particularly significant up-regulation during the last germination stage (phase IV). This group was mainly involved in carbohydrate metabolism and proteometabolism. In pattern C, 12 protein spots representing 10 DEPs displayed an up-down-up expression trend. These proteins were mainly related to basic metabolic activity such as carbohydrate metabolism and proteometabolism. Pattern D contained 15 protein spots (15 unique DEPs) that exhibited a down-regulated expression and decreased especially rapidly in phase IV; these proteins were mainly involved in carbohydrate metabolism and stress-related proteins. Pattern E displayed an up- to down-regulated expression trend containing 12 protein spots (10 unique DEPs).

Endosperm exhibited three main expression patterns (F-H) at protein level, as shown in Figure 4. Pattern F, with 6 protein spots representing 6 unique DEPs, displayed a down- to up-regulated expression trend, and proteins were mainly involved in carbohydrate metabolism and protein storage. In late germination, the DEPs in pattern F increased during mobilization of reserved substances. Pattern G showed an up-regulated expression trend, including 17 protein spots and 11 unique DEPs. On the contrary, pattern H included 11 protein spots and 9 unique DEPs and displayed a down-regulated expression trend, and proteins were mainly involved in storage and stress-related roles.

### Comparative proteome analysis of embryo and endosperm during different seed germination stages

**Figure 5:**
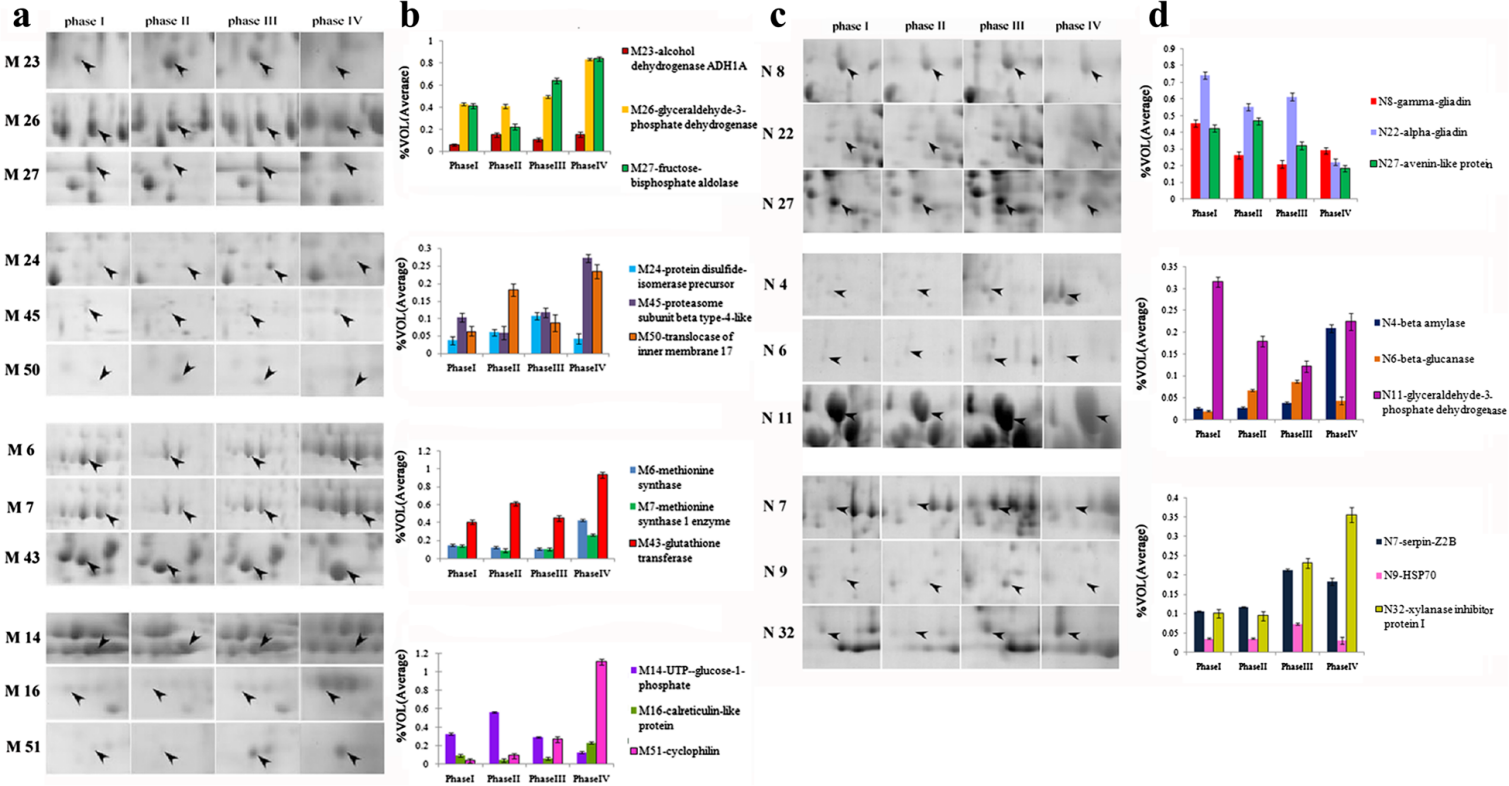
**Expression patterns of 21 important DEP spots from embryo (a, b) and endosperm (c, d) during seed germination. a**, **c**: The close-up view of the expression levels for 21 protein spots on 2-DE gels during four germination periods. **b**,** d**: The expression trends of the abundance (Vol %) of the 21 DEP spots.

The results listed in Additional file 4: Table S4 showed greater proteome differences during seed germination between embryo and endosperm. We compared the protein spots between both organs which were different spots only identified as the same protein name. Only eight common DEPs were present in both embryo and endosperm, and they were mainly responsible for carbohydrate metabolism, proteometabolism, amino acid metabolism, stress responsive proteins, and protease inhibitors. However, six of the proteins displayed different expression patterns, and only two DEPs (class II chitinase and proteasome subunit alpha type-3) showed the same expression trend during seed germination. Glyceraldehyde-3-phosphate dehydrogenase (GPD) (M26 and N11) was the first enzyme for which sequence data of the Calvin cycle/glycolysis homologues became available [28]. It was up-regulated (pattern B) in the seed embryo (Figure 5a-b) but had a down- to up-regulated expression (pattern F) in endosperm (Figure 5c-d). As an important physiologically active molecule involved in amino acid metabolism, methionine synthase (Met) (M6 and N2) showed down- to up-regulation (pattern B) and up-regulation (pattern G), respectively (Figures 4 and 5a-b). M54 exhibited pattern D in the embryo, N4 showed pattern G in the endosperm, and both were identified as beta-amylase, a major enzyme that hydrolyzes starch for glucose provision during late seed germination. In addition, M11 and N1 (dihydrolipoyl dehydrogenase 1), M66 and N17 (lactoylglutathione lyase), and M9 and N7 (Serpin-Z2B) also displayed different expression patterns in embryo and endosperm (Figure 4).

Figure 5a-b shows the expression patterns of 12 key DEP spots in the embryo that generally displayed up-regulated expression during seed germination and were mainly involved in basic metabolisms and stress response. Six DEP spots showed particularly significant up-regulation at late germination stages, especially at phase IV, including GPD (M26) and fructose-bisphosphate aldolase (M27), which were involved in carbohydrate metabolism, glutathione transferase (M43) related to amino acid metabolism, proteasome subunit beta type-4-like (M45), translocase of inner membrane 17 (TIM 17) (M50), and cyclophilin (M51) involved in proteometabolism. Five DEP spots exhibited a tender up-regulation during seed germination: alcohol dehydrogenase (ADH) (M23), protein disulfide-isomerase precursor (PDI) (M24), methionine synthase (M6), methionine synthase 1 enzyme (M7), and calreticulin-like protein (M16); however, UTP-glucose-1-phosphate (M14) showed an up-down expression pattern and had a higher expression level at stages I and II.

The expression patterns of nine key DEP spots in endosperm shown in Figure 5c-d were mainly related to storage proteins, carbohydrate metabolism, inhibitors, and stress response proteins. The storage proteins γ-gliadins (N8 and N25), α-gliadins (N14, N20, N22 and N24), gliadin/avenin-like seed proteins (N33 and N34), and avenin-like proteins (N12, 23and N27) were generally down-regulated during germination. Among carbohydrate metabolism, β-glucanase (β-Glu) (N3, N6) participated in the degradation of cell wall related to the mobilization of reserved substances and transportation and showed an up-down expression pattern (Figures 4 and 5c-d). Cytoplasmic aldolase (N13) as an important enzyme in glycolysis and dihydrolipoamide dehydrogenase (N5) as part of the multienzyme complex, which catalyzed pyruvic acid to acetyl-CoA, exhibited a down-up-regulation pattern during germination, as shown in Figure 4. Both xylanase inhibitor protein I (XIP-I) (N32) and serpin-Z2B (N7) displayed an up-regulated expression, particularly with a much higher expression level at stages III and IV (Figure 5c-d). Heat shock protein 70 (Hsp 70) (N9) was related to protein folding and participated in basic metabolic activity, and its expression abundance increased until phase III, then decreased (Figure 5c-d).

### Protein-protein interaction network analysis of the key DEPs

**Figure 6:**
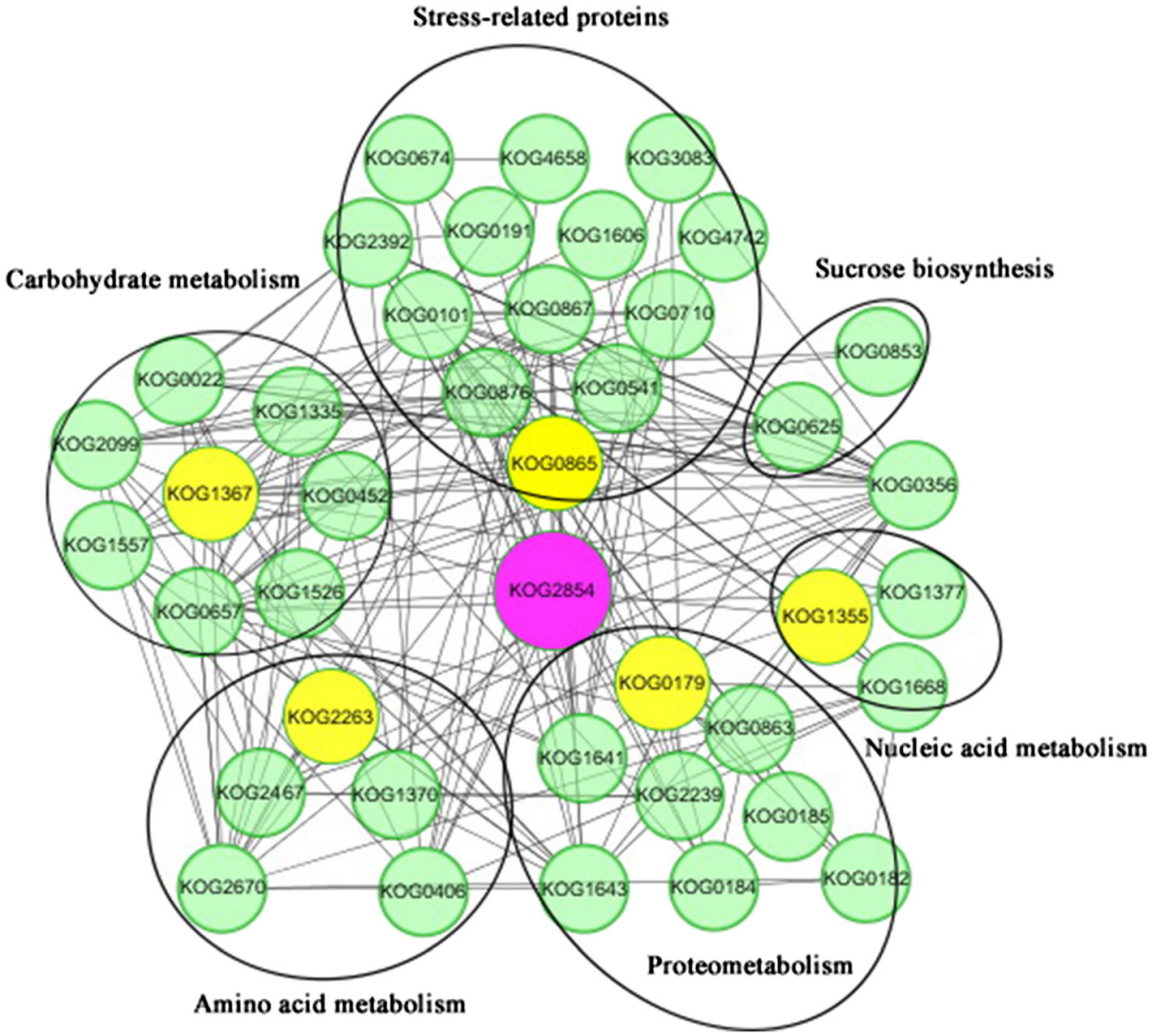
**Protein-protein interaction network of ATP synthase and related proteins.** Nodes with pink background color represent the KOGs of ATP synthase; nodes with yellow background represent the KOGs of main DEPs in each functional group; nodes with green background represent the KOGs of related DEPs in each functional group.

Our results showed that the endosperm had limited DEPs, mainly storage proteins and carbohydrate metabolism-related proteins (Figure 3) that could provide nutrition for germination and subsequent seedling growth, during the seed germination process (Figure 4). But in the embryo, considerable DEPs were identified during seed germination, including those involved in multiple metabolic processes such as stress response, carbohydrate metabolism, and proteometabolism (Figure 3). These DEPs could interact each other or with other proteins and function in seed germination. Thus, an interaction network of some key DEPs identified in the embryo was constructed using online analysis software STRING (version 9.1, http://string-db.org/). Proteins with different GI numbers may have the same KOG number, such as spots M6 and M7 sharing KOG2263 and M56 and M57 sharing KOG1370. All 47 KOGs listed in Additional file 5: Table S5, including 57 DEPs in the embryo, were displayed through Cytoscape (version 3.0, http://cytoscape.org/). To improve the reliability of this analysis, the confidence score was set to 0.700. As shown in Figure 6, six main interacting proteins were frequently located at the central part of the interaction network. KOG2854, which represents ATP synthase beta subunit (M67), was used to extract the potential interacting proteins from the whole protein-protein interaction network, and an ATP synthase beta subunit-centered sub-network was constructed (Figure 6) and showed that the ATP synthase beta subunit interact with 47 proteins involved in carbonmetabolism, amino acid metabolism, nucleic acid metabolism, proteometabolism, sucrose biosynthesis, and defense. The ATP synthase beta subunit aids ATP biosynthesis (energy metabolism) and is the universal enzyme that synthesizes ATP from ADP and phosphate using the energy stored in a transmembrane ion gradient [29-31].

### Transcriptional expression patterns of six key protein genes during seed germination

**Figure 7:**
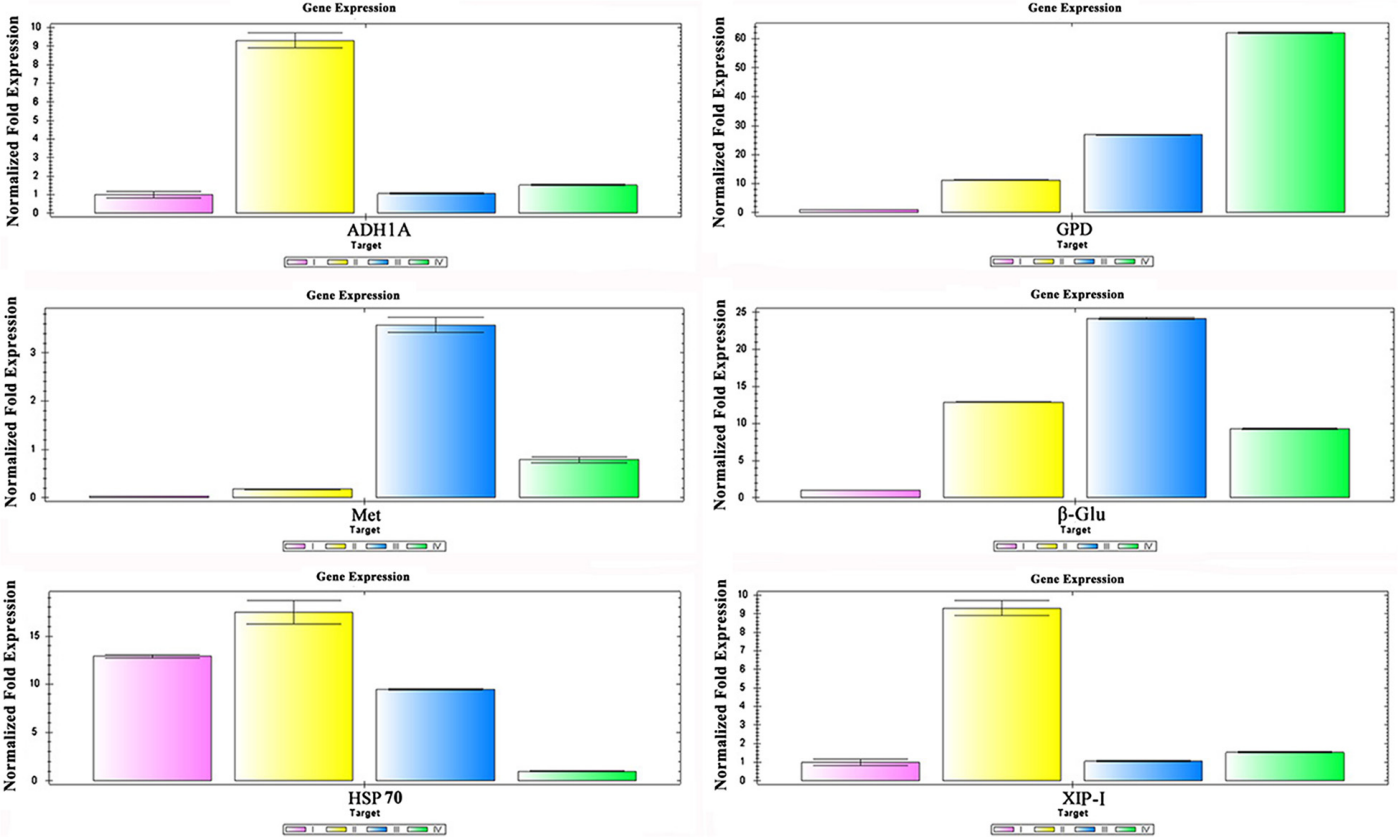
**The dynamic transcriptional expression changes of the genes encoded six key DEP spots during seed germination as revealed by qRT-PCR.** Three genes encoding alcohol dehydrogenase (ADH1A), glyceraldehyde-3-phosphate dehydrogenase (GPD), and methionine synthase (Met) were from embryo, and three genes encoding β-glucanase (β-Glu) and HSP 70 and xylanase Inhibitor protein-I (XIP-I) were from endosperm.

The transcriptional expression changes of the genes encoding six representative DEPs identified from wheat embryo and endosperm during seed germination are shown in Figure 7, including *ADH1A*, *GPD*, and *Met* from the embryo and *β-Glu*, *HSP70,* and *XIP-I* from the endosperm. Three types of expression patterns (A, B, and C) were recognized after careful analysis. In pattern A, the gene expression level was first up-regulated then down-regulated. Proteins encoded by genes corresponding to this pattern included *Met, β-Glu,* and *HSP 70*. Pattern B showed an up-regulated trend and included *XIP-I* and *GPD*. In pattern C, the *ADH1A* gene was first up-regulated, then down-regulated, and up-regulated in the last stage. A possible explanation is that when germination was complete, oxygen consumption in the embryo increased continuously but decreased in storage tissues because of the exhaustion of reserve substances or decreased dependence of seedlings on reserve substances. The expression patterns of all six genes were consistent with their protein expression trends (Figures 4 and 5).

## Discussion

### Energy requirements for seed germination

Respiration and energy production play key roles in whole seed germination. Seeds in the activation process begin soon after absorbing plenty of water. The activation sequence is amino acid metabolism, glycolysis, and the TCA cycle. Due to limited oxygen content, TCA cycle efficiency is not high in the early germination stages, and energy is supplied mainly by glycolysis and alcohol fermentation [23]. The energy provided by anaerobic respiration cannot satisfy the needs of germinated seeds at this point, but the TCA cycle also provides a large amount of energy in oxygen-rich conditions. The energy for seed germination is provided mostly by anaerobic respiration in the early stages, then respiratory activity increases as oxygen uptake and carbon dioxide release accelerate during imbibition [32]. The embryo and storage tissues of most seeds during germination exhibit a basic pattern of oxygen consumption [5]: they imbibe plenty of oxygen at the beginning of germination, after which they enter a lag period when the need for oxygen in the embryo is little. Oxygen consumption then increases sharply when the radicles break through the episperm, and germination is finished.

In the embryo, ADH (M23) is believed to participate in the glycometabolism pathway from pyranic acid to alcohol. The energy demands of germinating cereal seeds seem to be met mostly by glycolysis in the absence of oxygen [23]. It has been reported that ADH is involved in coleoptile growth under oxygen-limiting conditions, and rice coleoptile growth is depressed by deficiencies of ADH [33-36]. ADH increased from phase I to II and decreased in phase III in this study (Figure 5a-b); the variation trends correspond with the seeds’ pattern of oxygen consumption [5], an expression trend that was also found in dark-germinated pea seeds [37]. In addition, the gene expression of ADH at the transcription level also first increased, then decreased (Figure 7), consistent with the expression of ADH at the translation level.

GPD (M26) was a key enzyme during glycolysis, and its expression trend was up-regulated during all four seed germination stages (Figure 5a-b). The transcription expression of this enzyme corresponds well with its protein expression pattern (Figure 7). However, in the endosperm, GPD (N11) decreased quickly in the first three periods and increased when germination was complete (Figure 5c-d), so we can draw the conclusion that the speed of the glycolytic pathway in the seed embryo is slower than in the endosperm. In the process of seed germination, all kinds of stored substance (mainly in the endosperm) are broken down into a variety of small molecules, thus providing the respiration substrates as well as substance transformation and synthesis and a number of raw materials to form the new cells. Triosephosphat-isomerase (M40, M41) also participates in glycolysis. When germination begins, all embryo cells need more energy to form new organs such as the radicel and germ, so glycolysis increases and all enzymes related to glycolysis also increase. When germination is finished, consumption of reserve substances slows down and leads to slower glycolysis. Glycolysis is the dominant pathway for formation of pyruvic acid and ATP during germination. In our study, GPD always increased in protein expression and kept the same pattern at the transcriptional expression level during the seed germination process (Figures 5a-b and 7).

Aconitate hydratase (M1) exists in cells, not only in cytoplasm but also in mitochondria, and participates in the TCA cycle in mitochondria [38,39]. In dry seeds and seeds that imbibed early, mitochondrial structure and function were found to be defective [40]. Our observations showed that the structures of embryo cells in early germination were also defective (Figure 1d); therefore, the requirement of enzymes involved in the TCA cycle was low, and some of them were degraded during early germination such as dihydrolipoyl dehydrogenase 1 (M11) and alpha-glucan phosphorylase (M5). These enzymes displayed lower expression quantity in early germination stages (Figure 4). M5 is an enzyme that catalyzes the reversible phosphorylation reaction, converting malt sugar, starch, or glycogen into glucose-1-phosphate; therefore, it plays an important role in carbohydrate metabolism [41,42]. Its expression was an up-down-up trend, increasing especially rapidly in the last period (Figure 4), which corresponded with the pattern of oxygen consumption by the seeds [5]. In endosperm, dihydrolipoamide dehydrogenase (N5) is a component of the plastidial pyruvate dehydrogenase complex, which participated in the preparation for pyruvic acid entering TCA cycle. Its expression trend first decreased then increased (Figure 4), suggesting that the pattern of oxygen consumption by seed endosperm may be in accord with the basic oxygen consumption pattern by seed. Correspondingly, due to the continuous synthesis of pyruvate, which was turned into alcohol and entered the fermentation pathway, pyruvic acid could be further degraded through the TCA cycle. Thus, most TCA cycle-related proteins identified in this work, such as aconitate hydratase (M1), dihydrolipoyl dehydrogenase 1 (M11), and alpha-glucan phosphorylase (M5) (Figure 4), were generally up-regulated.

When the germination is complete, and the one promoting factor (GA) produced by the embryo induced certain endosperm cells to express a number of enzymes involved in the mobilization of most storage proteins, starch, and triacylglycerol (the main lipid of seed) from the endosperm [43,44]. In fact, most of these enzymes existed in the endosperm in the first place, but they were inactive until they were activated or re-synthesized prior to mobilization [5,45]. At this time, most reserve substances from endosperm began to be mobilized to supply nutriments for seedling growth, so only a small amount was degraded during the germination process [46].

In the late germination stages, the hydrolysis of some storage proteins produces amino acids and peptides that may either remain in the endosperm or be transformed into Asn or Gln, then transported to the seedlings [47]. Most storage proteins, such as γ-gliadin (N25), α-gliadin (N22 and N24), gliadin/avenin-like seed protein (N33 and N34), and avenin-like protein (N23 and N27), are degraded when germination is complete (Figure 4). This phenomenon demonstrated that the main storage proteins were mobilized after germination at the molecular level.

### Starch and sucrose metabolism

Starch is the major reserve in mature cereal seed. Wheat seeds contain about 70% starch and 10% storage proteins. The degradation of these materials provides energy for seed germination and seedling growth. When the seed endosperm imbibes a certain amount of water, starch mobilization occurrs after deposited sucrose degradation. The mobilization of starch is a complex process including the lysis of endosperm cells wall, starch hydrolysis, and the synthesis or transport of sucrose. *β*-glucanase (N3 and N6), one kind of hemicellulase, which participates in the degradation of cell wall, first increased then decreased during seed germination in this study (Figure 5c-d), which corresponded well with its transcriptional expression pattern (Figure 7). The consistency of *β*-glucanase gene expression at transcriptional and translational levels confirmed that the mobilization of starch happened during germination at the molecular level. The decomposition products of triacylglycerol were glycerol and fatty acid (FA). FA could be oxidized and used for respiratory metabolism, and glycerol could be not only oxidized by TCA cycle but also transformed into glucose by a suite of metabolic reactions [48,49]. Then sucrose was synthesized from glucoses and transported to seedlings in the form of sucrose. Beta-amylase (N4) increased gradually in the first three germination periods and increased dramatically to about four times during the fourth period (Figure 5c-d), suggesting that starch degradation metabolism is vigorous at the end of seed germination. Similar results were also found in the germination of barley seeds [50]. These results imply that large-scale starch mobilization might happen during the late germination stage and after germination to provide nutriments for subsequent seedling growth. Beta-amylase is a major enzyme that hydrolyzes starch for glucose provision during late germination. We can conclude that starch hydrolysis becomes more and more intense during the germination process. Starch is converted to glucose after a series of catabolism, and sucrose is further synthesized, then transported to the seedlings for growth and development. Previous work found that the majority of sucrose resulted from storage lipid degradation, not from other soluble sugars within *Arabidopsis* seeds [15]; however, our results revealed that the majority of sucrose comes from the mobilization of storage starch and lipids, similar to germinating barley seeds [44]. Sucrose may be resolved into glucose and fructose by sucrose synthase, then enter glycolysis. We found that sucrose synthase type 2 (M52) increased in the latter germination stages for seedling growth (Additional file 4: Table S4).

### Protein metabolism

Besides starch, the other most important energy reserves are seed proteins, especially storage proteins. Dry seeds of wheat have various kinds of enzymes or proteins, but most of them have no activity. When seeds begin to germinate, they could participate in a series of basic metabolic activities immediately, such as energy metabolism and synthesis of proteins. Dry seeds contain not only the above proteins but also numerous components related to the synthesis of proteins [51]. Once the dry grains contact water, mRNAs and ribosomes assemble rapidly into polysomes, then immediately begin to synthesize proteins with other components [52]. During early germination, the RNA required for protein synthesis exists in dry seeds and supports the synthesis of early germination proteins, including proteins concerned with basic metabolic activity and germination [53]. In addition to early proteins, other proteins are degraded into amino acids for early protein synthesis. As germination progresses, protein synthesis becomes increasingly dependent on the synthesis of mRNA, the compositions of which are changing, and then the types of proteins expressed in the embryo change [54].

Proteasome (M35, M42, M45, and N30), a large protein aggregate with several proteolytic activities, has 7 different *α* and *β* subunits, respectively, in eucaryon [55] and participated in proteolysis. Proteins are marked for degradation by the proteasome when they are modified by the addition of ubiquitin [56]. PDI (M24) and TIM 17 (M50) are associated with the transport and processing of protein, and their expression is mainly up-regulated (Figure 5a-b). PDI (M24) is an essential protein that facilitates the isomerization of disulfide bonds [57], especially in assisting the folding of synthesized proteins [58]. TIM 17 was concerned with protein transport. These two enzymes from the embryo participate in basic metabolic activity, so they increase as germination begins. In endosperm, proteins are catabolized upon the initiation of germination by hydrolytic enzymes secreted from the aleurone layer. The hydrolyzed storage proteins act as an energy source, providing carbon and nitrogen for seed germination and subsequent seedling establishment. Most storage proteins, such as γ-gliadins (N8 and N25), α-gliadins (N14, 20, 22 and N24), gliadin/avenin-like seed proteins (N33 and N34), and avenin-like proteins (N12, 23 and N27), are down-regulated after germination (Figure 4).

### Amino acid and nucleic acid metabolism

Met (M6,M7) is an important amino acid metabolism enzyme. It catalyzes the transfer of a methyl group from methyl-cobalamin to homocysteine [59]. The expression of this enzyme decreased in early germination then increased, as shown in Figure 5a-b. The expression of M6 quantity increased to its maximum during the fourth period, an expression trend implying that these were not early germination proteins. The expression of the Met gene at the transcriptional level was generally up-regulated and achieved its peak expression in the third period (Figure 7). Protein translation is known to lag behind the transcriptional expression of protein, so gene expression at the transcription and protein level was also consistent. In the endosperm, Met (N2) content increased throughout germination, suggesting that it is the early germination protein in endosperm and undergoes protein degradation. Hence, the embryo is in the process of protein synthesis at this moment, but the endosperm undergoes protein degradation. The metabolism of nucleotides was not active at early stages of germination because few enzymes required for nucleotide metabolism were detected. Both UTP--glucose-1-phosphate (M14) and adenylosuccinate synthetase (M21) were nucleic acid metabolism proteins, which play a central role as glucosyl donors in cellular metabolic pathways. Adenylosuccinate synthetase (AdSS) plays an important role in the de novo synthesis of adenylmonophosphate and in the salvage pathway of purine nucleotide biosynthesis [60]; its express trend is a down-up-down pattern (Figure 4), and a possible reason is injured RNA (especially mRNA) molecules in dry seeds (phase I) inside the cell membrane system. As the water absorps, cells begin repair activities, and new RNA is constantly formed to synthesize various enzymes. When germination is complete (phase IV), RNA synthesis decreases, so Adss content dropped significantly.

### Stress-related proteins mobilization

Due to changes in external environments, wheat seeds are able to activate a series of mechanisms to respond to many biotic and abiotic stresses during germination [61]. Calreticulin-like protein (M16 and M13) is an abundant Ca^2+^-binding protein predominantly located in the endoplasmic reticulum (ER) of higher plants. It is a key player in plant growth and development, a powerful regulator of plant responses to various stresses, and can help assemble other proteins correctly [62]. It also serves as a calcium-binding protein in the endoplasmic reticulum with functions like lectin combined with molecular calcium [63-65]. Its expression trend was mainly up-regulated, especially in the last period of germination (Figure 5a-b), which hints that wheat seed may encounter stress in late germination [66]. Cyclophilin (M51) has multiple biological activities involved in signal transduction, cell apoptosis, and protein folding. It is highly conservative and possesses peptidyl-proly cis-trans isomerase activity [67]. In higher plants, cyclophilin genes from *Zea mays* and *Brassica napus* were separated and expressed [68]. Our results show that M51 increased slowly in first three germination stages but increased sharply in the last stage (Figure 5a-b). Because the cellular structure is not complete in early germination, metabolic activities such as protein synthesis are low, so cyclophilin expression is very low. In the following three phases, however, it increased significantly along with the perfection of the cellular structure and the increase of metabolic activities in germinating seeds.

Reactive oxygen species (ROS) are produced in all living organisms, and their overaccumulation of ROS can result in oxidative stress. Reducing oxidized proteins is another critical way for plants to cope with ROS. Upon imbibition, ROS contents increased gradually. ROS can be scavenged efficiently by the antioxidant enzymes such as superoxide dismutases (SODs) (M47 and M71), glutathione S-transferase (GST) (M69), and peroxidases (M72,N19 and N21). Many of these redox regulation proteins were identified during germination [69]. In this study, these proteins showed gradually up-regulated expression when germination began (Figure 4). All of them can reduce the target proteins’ disulfide and keep these proteins functional [70-73]. This result indicates that wheat may prefer to protect functional proteins from ROS attack during seed germination.

Stress responsive proteins, including HSPs and late embryogenesis abundant (LEA) proteins (M56), accumulated during seed maturation [23,24]. HSPs participate in diverse cellular processes by acting as molecular chaperones. They belong to a huge glycoprotein gene family, of which HSP 70 (N9) is the most conservative [74]. They also are described as being developmentally regulated, abundant in dry mature seeds, and disappearing during germination [75]. In agreement with these findings, the expression of Hsp70 (N9) at both transcriptional and translational levels increased at phase III and decreased by the end of the germination process (Figures 5c-d and 7) in accord with the synthesis of plentiful enzymes that participated in the mobilization of reserve substances. Its down-regulation after germination suggests that the metabolic activity of endosperm weakened when accompanied by the large consumption of reserve substances.

### Other protein metabolisms

According to our results, the photosynthesis process was mobilized slightly after initiation of reserve mobilization during seed germination, indicating that subsequent (after 35 hours of imbibition) growth depends not only on mobilization of energy reserves but also on photosynthesis. Ribulose-1,5-bisphosphate carboxylase/oxygenase (RuBisCO, M74) increased rapidly in the late germination stages (Additional file 4: Table S4). It methylates 'Lys-14' of the large subunit of RuBisCO and also can use chloroplastic fructose-bisphosphate aldolases with lower efficiency [76]. So we can conclude that the photosynthetic apparatus was gradually integrated into the seed in late germination.

The ATP synthase beta subunit (M67) contributes to catalytic sites that bind ATP and ADP. It plays a pivotal role in energy transduction in living cells [77]. Its expression trend showed an up-regulated pattern (Figure 4) and indicated that energy metabolism continuously strengthened the process of germination. Translation elongation factor 1-beta (M33) stimulates the exchange of GDP bound to elongation factor-1-alpha to GTP and has actual GDP/GTP exchange activity [78]. It displayed a generally up-regulated trend (Figure 4), especially in the last period. We speculate that new protein synthesis is more active when germination is complete, and this may be related to the emergence of germs and radicles. Pyridoxine biosynthesis protein (M31) is a limited velocity enzyme in the process of vitamin B6 synthesis. Studies have shown that it relates to the plasma membrane and inner membrane systems. Vitamin B6 is related to membrane system repairs in early germination and may play an important role in the function of the membrane [79]. Its expression pattern in this study (M31) was up-down-regulated. Its expression decreased significantly when germination was finished (Figure 4). So we can know that seed undergoes membrane system repairs in early germination and related enzymes content will increase accordingly. In addition, 27 K protein (N31) as a glycoprotein with mannose and fucose residues may belong to the family of γ-interferon-inducible thiol reductases. It is also a kind of serine/arginine-rich (SR) protein and could modulate pre-mRNA splicing reaction [80]. Its expression showed a down-regulated pattern (Figure 4), suggesting that the synthesis of mRNA decreased greatly in the late germination stages.

### Overview of central metabolic pathways during wheat seed germination

**Figure 8:**
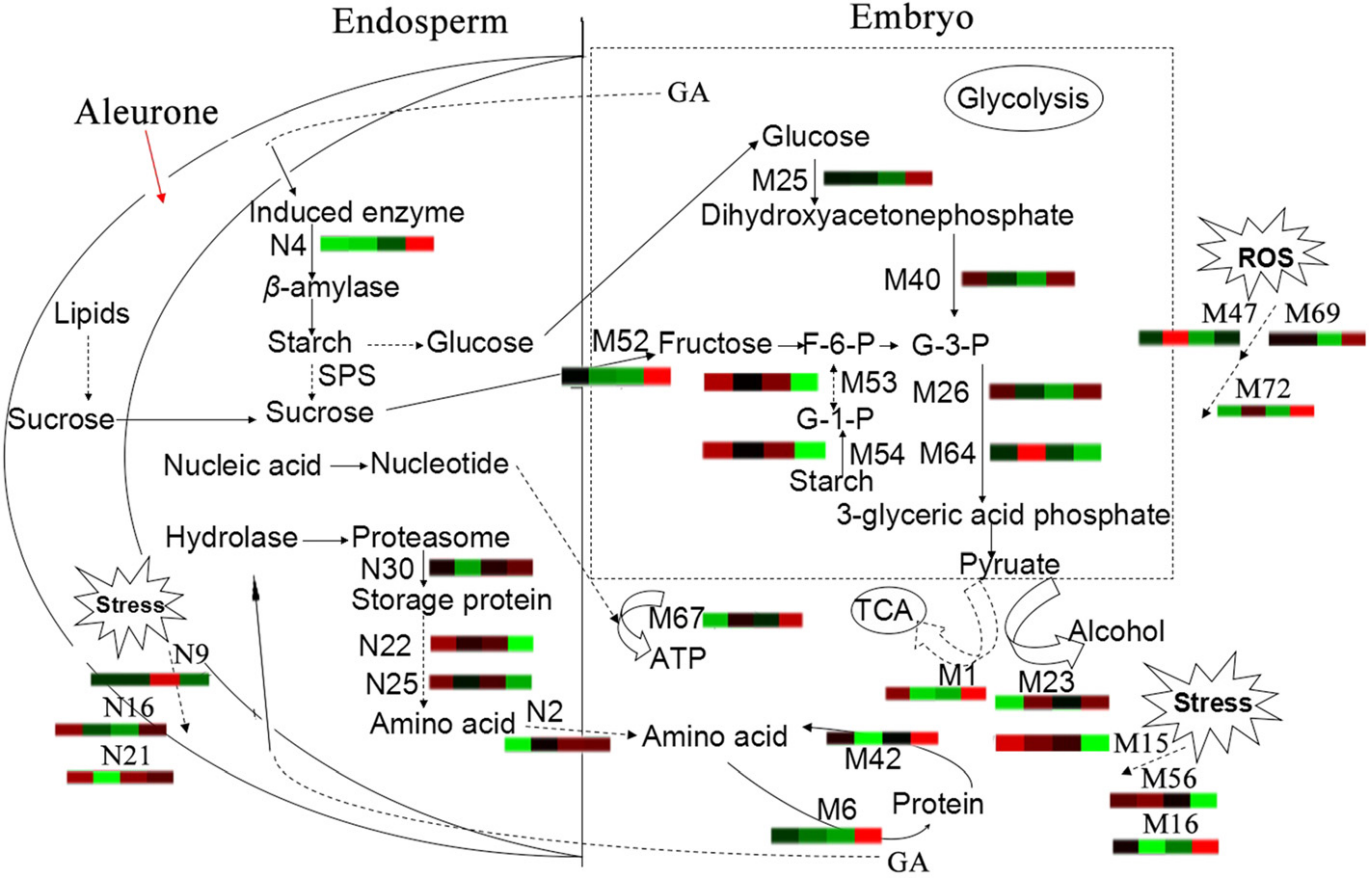
**A putative metabolic pathway of wheat embryo and endosperm during the germination process.** M1: putative aconitate hydratase; M6: methionine synthase; M15: Em protein H5; M16: calreticulin-like protein; M21: adenylosuccinate synthetase; M23, alcohol dehydrogenase ADH1A; M25: sucrose synthase type 2; M26: glyceraldehyde-3-phosphate dehydrogenase; M40: triosephosphat-isomerase; M42: proteasome; M47: manganese superoxide dismutase; M52: sucrose synthase type 2; M53: phosphoglucomutase; M54: Beta-amylase; M56: putative late embryogenesis abundant protein; M64: cytosolic 3-phosphoglycerate kinase; M67: adenosine kinase 2; M69: glutathione S-transferase; M72: peroxiredoxin-2E-1; N2: methionine synthase; N4: beta amylase; N9: HSP70; N16: class II chitinase; N21: class II chitinase; N22: alpha gliadin; N25: gamma- gliadin; N27: avenin-like protein; N30: proteasome subunit alpha type-3; SPS: Suc phosphate synthase; F-6-P: fructose-6-P; G-6-P: glucose-6-P; G-1-P: glucose-1-P; G-3-P: glyceraldehyde-3-phosphate.

To better understand the central proteomic changes of embryo and endosperm during wheat seed germination, a putative metabolic pathway was proposed, as shown in Figure 8. In the embryo, the activation process begins soon after seeds absorb plenty of water. The activation sequence is amino acid metabolism, glycolysis, and the TCA cycle. The energy demands of germinating cereal seeds seem to be met mainly by glycolysis [23]. The TCA cycle also provides a large amount of energy in oxygen-rich conditions. Many important related proteins are involved in various metabolic pathways. The embryo synthesizes and secretes GA that is transferred to the aleurone layer of the endosperm. GA then induces the synthesis of β-amylase in the aleurone layer, which secretes hydrolysis enzymes to the starchy endosperm [2]. The embryo utilizes sugars released by starch degradation for its growth. In the endosperm, all storage proteins showed a down-regulated expression trend and were broken into small molecules and used for the development of the embryo and seedling growth. Some stress-related proteins and ROS scavenging proteins were activated and displayed an up-regulated pattern. Upon imbibition, the contents of ROS increased gradually, resulting in further oxidative stress. ROS can be scavenged efficiently by antioxidant enzymes such as SODs (M47), GST (M69), and peroxidases (M72). The cooperative functions of these important proteins provide a sound basis for regular germination of seed and subsequent seedling growth.

## Conclusion

In this study, 74 and 34 DEP spots representing 63 and 26 unique proteins were identified from embryo and endosperm, respectively, during seed germination. Seed embryo and endosperm display distinct differentially expressed proteins involved in seed germination; in particular, DEPs from the embryo were mainly related to carbohydrate metabolism, proteometabolism, amino acid metabolism, nucleic acid metabolism, and stress response, whereas those from the endosperm were mainly involved in storage proteins, carbohydrate metabolism, stress response, and protein synthesis. During germination, the embryo and endosperm of wheat seeds possibly have a basic pattern of oxygen consumption. They consume plenty of oxygen at the beginning of germination, enter lag period, then oxygen consumption increases sharply when the radicles have broken through the episperm. When germination is complete, oxygen consumption increases continuously in the embryo but decreases in the endosperm. Reserve substances from the endosperm begin to be mobilized when the germination is finished, and more enzymes involved in these processes are synthesized in large quantities. The mobilization also possibly concerns the activation of enzymes and inactivation of inhibitors. Storage proteins produce amino acids, peptides, and their derivatives, which are used to synthesize new proteins or transformed into Asn or Gln, then transported to the seedling. A putative metabolic pathway was proposed, which displays the central proteomic changes of embryo and endosperm during wheat seed germination.

## Methods

### Plant material

The elite Chinese bread wheat (*Triticum aestivum* L.) cultivar Zhengmai 9023, which has high yield, superior gluten quality, and good biotic resistance and has been widely cultivated in the main Chinese wheat production areas during the past ten years, was used in this study [81].

### Seed germination and sampling

Seeds with similar sizes and weights were selected as the experimental materials and washed with distilled water three times. Seeds were germinated on wet filter paper in Petri dishes and incubated at 25°C in a growth chamber in the dark. Imbibed seeds can progressively lose dormancy in a matter of weeks if they are kept in constant darkness [82]. Water was added as needed to ensure complete imbibitions and normal germination. To understand the protein trends in the wheat embryo and endosperm, our study investigated four periods of the seeds germination, including phase I (seeds absorbing water for 5 min), phase II (seeds absorbing water for 5 hours), phase III (radicles broke through the episperm and seeds will sprout soon), and phase IV (green germ and three radicles visible). The embryo and endosperm collected from four germination periods with three biological replicates were separated and stored at −80°C prior to use.

### Light microscopy observation

Transverse slices approximately 1 mm thick were cut from the collected wheat embryo and endosperms during each of the four periods and were fixed, rinsed, dehydrated, infiltrated, and polymerized by the series of steps outlined in Arcalis et al. [83]. For light microscopy observation, sections approximately 800 nm thick were cut, collected on mesh nickel grids, and stained with toluidine blue. Because the seed embryo from phase IV exhibited a large change compared with the first three phases, its cell structures were not observed by light microscope.

### Protein extraction

About 0.5 g of wheat embryo and endosperm were ground into a fine powder in liquid nitrogen [84]. After 1 ml of extraction buffer (0.25 M sucrose, 1 M pH7.5 Tris-HCl, 0.1 M EDTA) was added into each sample, 10 μl 0.1 M PMSF and 0.1 M DTT were added immediately. Then the samples were ground for 10 min and 1 ml extraction buffer (0.25 M sucrose, 1 M pH7.5 Tris-HCl, 0.1 M EDTA, 4% (v/v) Triton-100), 10 μl 0.1 M PMSF, and 0.1 M DTT were added, and samples were ground again for 3 min. Then samples were transferred into tubes and vortexed for 10 min at room temperature. After centrifugation for 10 min at 13,000 rpm and 4°C, the supernatant was transferred into new tubes. This step was repeated twice, and the proteins in the supernatant were precipitated by adding 1/4 volumes 50% TCA buffer at -20°C for 2 to 3 hours, followed by centrifugation for 5 min at 13000 rpm and 4°C. The precipitate was washed three times with 1 ml chilled (-20°C) acetone containing 0.002 g DTT and centrifuged at 8,000 rpm and 4°C for 10 min between rinses. The fluid was removed and the pellet was dried under room temperature. Then the pellet was dissolved in 400 μl Lysis buffer (7 M urea, 2 M thiourea, 4% CHAPS) overnight. Finally, the protein concentration was determined with a 2-D Quant Kit (Amersham Bioscience, USA) using BSA (2 mg/ml) as the standard.

### 2-DE and image analysis

Each protein sample extracted from wheat embryo and endosperm (each about 600 μg), rehydration buffer (8 M urea, 2% w/v CHAPS), 0.5% (v/v) IPG buffer pH 3–10, 1% (w/v) DTT, and 1 μl 1% bromphenol blue were mixed to a final volume of 360 μl. The final solution was loaded onto the IEF linear IPG strips (pH 3-10, 18 cm, GE Healthcare) and actively rehydrated at 30 V for 12 h at 20°C in a Multiphor II apparatus (GE Healthcare). Focusing was performed on the IPGphor apparatus under the following conditions: 300 V for 1 hour, 500 V for 1 hour, 1000 V for 1 hour, 3000 V for 1 hour, and finally run at 8000 V for 80 kV h. After the first-dimension IEF, the IPG strips were equilibrated according to Gao et al. [85], then separated by 12% SDS-PAGE. After electrophoresis, gels were stained by colloidal Coomassie Brilliant blue (CBB) according to R-250/G-250 = 4:1. The images were visualized using a BIO-RAD GS-800 scanner. Protein spots that showed statistically significant changes by Student’s *t*-test (abundant difference at least 2-fold, P < 0.05) between samples from different germination stages and organs were determined as DEP spots by an ImageMasterTM 2D 5.0 software. After image analysis, corresponding DEP spots of interest were selected for tandem mass spectrometry analysis.

### MALDI-TOF/TOF-MS and protein identification

Protein spots were excised from the preparative gels and destained with 100 mM NH_4_HCO_3_ in 30% ACN. After removing the destaining buffer, the gel pieces were lyophilized and rehydrated in 30 μL of 50 mM NH_4_HCO_3_ containing 50 ng trypsin (sequencing grade; Promega, Madison, WI, USA). After overnight digestion at 37°C, the peptides were extracted three times with 0.1% TFA in 60% ACN. Extracts were pooled together and lyophilized. The resulting lyophilized tryptic peptides were kept at -80°C prior to mass spectrometric analysis. A protein-free gel piece was treated as above and used as a control to identify autoproteolysis products derived from trypsin.

MS/MS spectra were obtained using the ABI 4800 Proteomics Analyzer MALDI-TOF/TOF (Applied Biosystems, Foster City, CA, USA) operating in a result-dependent acquisition mode. Peptide mass maps were acquired in positive ion reflector mode (20 kV accelerating voltage) with 1000 laser shots per spectrum. Monoisotopic peak masses were automatically determined within the mass range 800–4000 Da with a signal to noise ratio minimum set to 10 and a local noise window width of m/z 250. Up to ten of the most intense ion peaks (minimum signal noise ratio: 50) were selected as precursors for MS/MS acquisition, excluding common trypsin autolysis peaks and matrix ion signals. In MS/MS-positive ion mode, spectra were averaged, collision energy was 2 kV, and default calibration was set. Monoisotopic peak masses were automatically determined with a signal to noise ratio minimum set to 5 and a local noise window width of m/z 250. The MS/MS spectra were searched against the NCBI nonredundant green plant and Triticum_ncbi database using GPS Explorer software, version 3.6 (Applied Biosystems) and MASCOT version 2.1 (Matrix Science) with the following parameter settings: trypsin cleavage, one missed cleavage allowed, carbamidomethylation set as fixed modification, oxidation of methionines allowed as variable modification, peptide mass tolerance set to 100 ppm, fragment tolerance set to ± 0.3 Da, total ion score confidence interval percentage and protein score confidence interval percentage were both set above 95%, and the significance threshold was p < 0.05 for the MS/MS.

### Quantitative real-time polymerase chain reaction (qRT-PCR)

The wheat embryo and endosperm samples from four germination periods were ground into fine powder in liquid nitrogen. Then, total RNA was extracted from each sample and reverse-transcribed into cDNA according to the procedures in Yang et al. [86]. Gene-specific primers were designed using online Primer3Plus (http://primer3.ut.ee/) according to Untergasser et al. [87]. The sequence of each protein was first used as a BLASTP search term against *Triticum aestivum* genome. The best aligned gene was used for primer design. The specificity of the primers was checked by observing the melting curve of the RT-PCR products and the specific band on agarose gel. The primer sequences for qRT-PCR assays are given in Additional file 6: Table S6. ADP was chosen as an internal control for data normalization. qRT-PCR was performed in a 20-μl volume containing 10 μl 2 × SYBR Premix Ex Taq™ (TaKaRa), 2 μl 50-fold diluted cDNA, 0.15 μl of each gene-specific primer, and 8 μl ddH_2_O. The PCR conditions were as follows: 95°C for 3 min, 40 cycles of 15 s at 95°C, 61°C for 30 s. Three replicates were used for each sample. Reaction was conducted on a CFX96 Real-Time PCR Detection System (Bio-Rad). All data were analyzed using the CFX Manager Software (Bio-Rad) and the 2 (-Delta Delta C(T)) method [88]. QRT-PCR efficiency was determined by five serial five-fold dilutions of cDNA, and the standard curve confirmed them at high RT-PCR efficiency rates (Additional file 7: Figure S2).

## Additional files

Additional file 1: Figure S1. The DEP spots and unique proteins identified in wheat embryo and endosperm during seed germination. Additional file 2: Table S1. The DEP spots and their functions from embryo and endosperm during seed germination identified by linear 2-DE (pH 3-10 and 18 cm strips) and MALDI-TOF/TOF mass spectrometry. **Table S2.** The unnamed and predicted proteins from embryo and endosperm in Table S1 grouped by BLASTP (www.ncbi.nlm.nih.gov/BLAST/) in searching for closest homologue. Additional file 3: Table S3. The peptide sequences of identified DEP spots in embryo and endosperm identified by MALDI-TOF/TOF-MS. Additional file 4: Table S4. Functions and expression features of some important DEPs in wheat embryo and endosperm. Additional file 5: Table S5. Eukaryotic orthologous groups (KOGs) of the key DEPs identified in the wheat embryo. Additional file 6: Table S6. The specific primer sequences for qRT-PCR assays. Additional file 7: Figure S2. qRT-PCR optimization design: double standard curve (a) and dissolution curve (b) of six genes in embryo and endosperm. The dissolution curves of different genes are indicated.

## Abbreviations

ADH: Alcohol dehydrogenase
AdSS: Adenylosuccinate synthetase
β-Glu: β- glucanase
2-DE: Two-dimensional electrophoresis
CBB: Colloidal Coomassie Brilliant blue
CRT: Calreticulin-like protein
DEP: Different expressed protein
DEPs: Differentially expressed protein spots
FA: Fatty acid
GPD: Glyceraldehyde-3-phosphate dehydrogenase
GA: Gibberellic acid
GST: Glutathione S-transferase
HSP 70: Heat shock protein 70
IEF: Isoelectric focusing
IPG: Immobiline™ DryStrip gel
Met: Methionine synthase
MALDI-TOF/TOF-MS: Matrix assisted laser desorption ionization/time of flight/tandem mass spectrometry
M: Embryo
N: Endosperm
PDI: Protein disulfide-isomerase
qRT-PCR: Quantitative real-time polymerase chain reaction
ROS: Reactive oxygen species
SODs: Superoxide dismutases
TCA cycle: Tricarboxylicacid cycle
TIM 17: Translocase of inner membrane 17
XIP-I: Xylanase inhibitor protein I
