# Supplementary figures and images for: Comparative proteome analysis of embryo and endosperm reveals central differential expression proteins involved in wheat seed germination
Source: BMC Plant Biol. 2015 Apr 8;15:97. doi: 10.1186/s12870-015-0471-z (PMC4407426; doi:10.1186/s12870-015-0471-z)

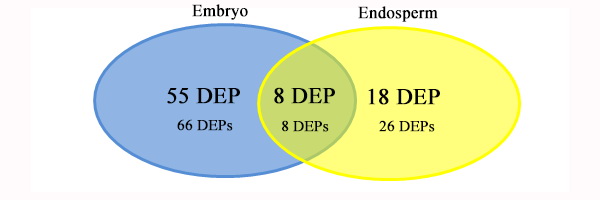

Supplement: Additional file 1: Figure S1. — The DEP spots and unique proteins identified in wheat embryo and endosperm during seed germination. [file 12870_2015_471_MOESM1_ESM.jpg]

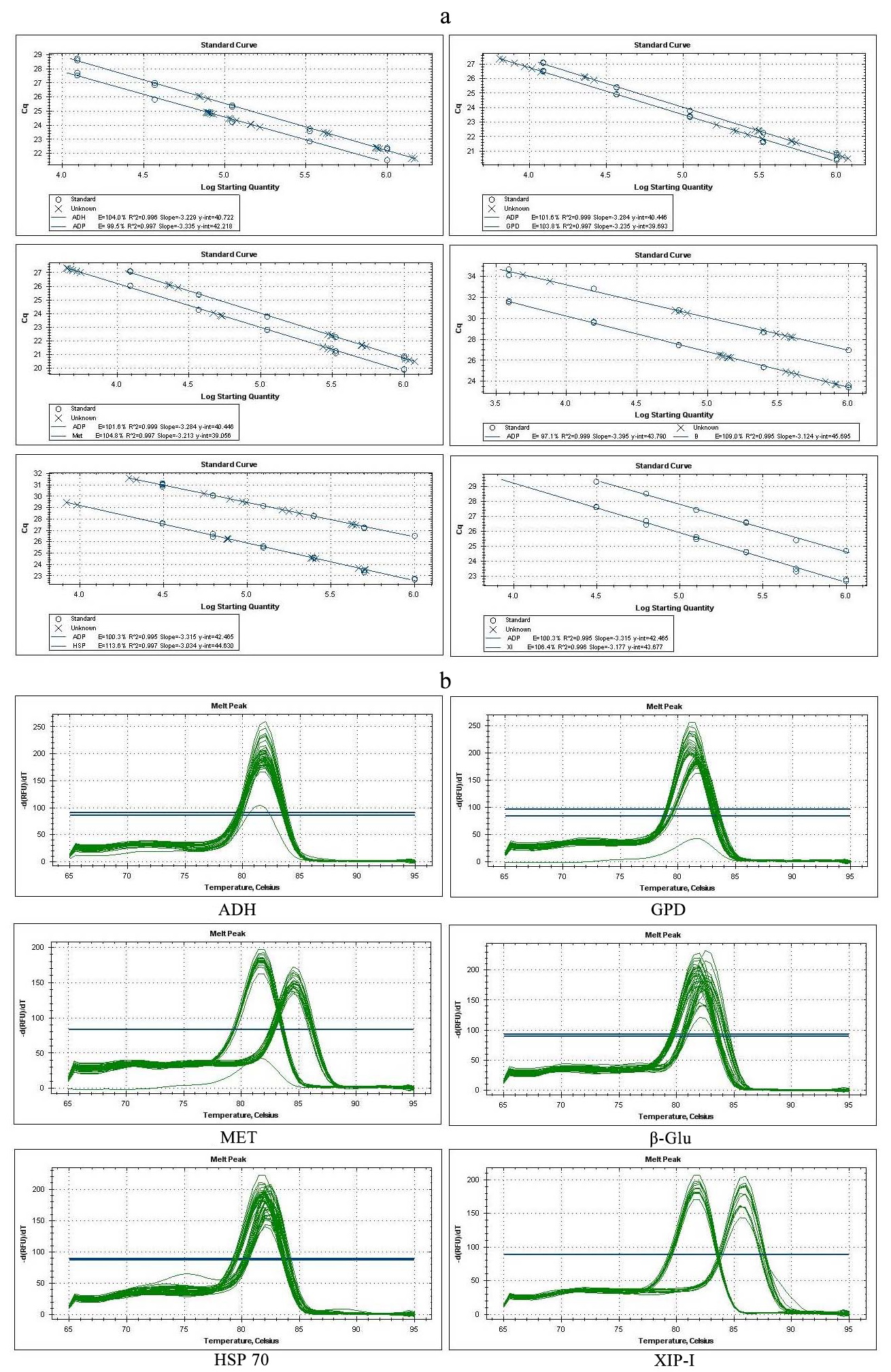

Supplement: Additional file 7: Figure S2. — qRT-PCR optimization design: double standard curve (a) and dissolution curve (b) of six genes in embryo and endosperm. The dissolution curves of different genes are indicated. [file 12870_2015_471_MOESM7_ESM.jpeg]
