# Supplementary material for: Comparative proteome analysis of embryo and endosperm reveals central differential expression proteins involved in wheat seed germination
Source: BMC Plant Biol. 2015 Apr 8;15:97. doi: 10.1186/s12870-015-0471-z (PMC4407426; doi:10.1186/s12870-015-0471-z)
Supplement: Additional file 2: Table S1. — The DEP spots and their functions from embryo and endosperm during seed germination identified by linear 2-DE (pH 3-10 and 18 cm strips) and MALDI-TOF/TOF mass spectrometry. Table S2. The unnamed and predicted proteins from embryo and endosperm in Table S1 grouped by BLASTP (www.ncbi.nlm.nih.gov/BLAST/) in searching for closest homologue. [file 12870_2015_471_MOESM2_ESM.docx]

**Additional file 2: Table S1** Proteins from embryo and endosperm differentially expressed during the seed germination and identified by linear 2-DE (pH 3-10 and 18 cm strips) and MALDI-TOF/TOF mass spectrometry.

| **Spot No.** | **Protein Name** | **Accsession No.** | **Species** | **Pep.**  **Count^a^** | **Protein Score^b^** | **Protein**  **Score C.I.%** | **Total Ion C.I.%** | **Average volume ratio (I:II:III:IV)** | **P-value** | **TpI/TMW^c^ (kDa)** | **EpI/EMW^d^ (kDa)** |
| --- | --- | --- | --- | --- | --- | --- | --- | --- | --- | --- | --- |
| **Embryo** |  |  |  |  |  |  |  |  |  |  |  |
|  | **Carbohydrate metabolism** |  |  |  |  |  |  |  |  |  |  |
| 1 | Putative aconitate hydratase | gi\|357144707 | *Oryza sativa subsp. japonica* | 20 | 409 | 100 | 100 | 1:0.4:0.4:1.4 | 0.012 | 5.76/99.00 | 6.45/99.10 |
| 5 | Alpha-glucan phosphorylase, | gi\|300681424 | *[Triticum aestivum](http://www.uniprot.org/taxonomy/4565)* | 36 | 601 | 100 | 100 | 1:2.1:1.1:6.8 | 0.026 | 7.6/94.13 | 7.74/85.0 |
| 11 | Dihydrolipoyl dehydrogenase 1 | gi\|357134512 | *Arabidopsis thaliana* | 16 | 253 | 100 | 100 | 1:0.7:0.6:1.4 | 0.017 | 6.93/53.07 | 6.82/66.50 |
| 23 | Alcohol dehydrogenase ADH1A | gi\|119388731 | *Triticum turgidum subsp.dicoccon* | 12 | 220 | 100 | 100 | 1:2.5:1.8:2.6 | 0.023 | 6.15/41.73 | 6.88/48.0 |
| 25 | Fructose-bisphosphate aldolase | gi\|226316439 | *Secale cereale* | 10 | 128 | 100 | 98.822 | 1:1:0.8:1.6 | 0.008 | 6.39/39.23 | 6.45/40.80 |
| 26 | Glyceraldehyde-3-phosphate dehydrogenase | gi\|253783729 | *Triticum aestivum* | 11 | 345 | 100 | 100 | 1:0.9:1.2:1.9 | 0.032 | 6.67/36.62 | 7.45/42.88 |
| 27 | Fructose-bisphosphate aldolase | gi\|226316439 | *Secale cereale* | 11 | 212 | 100 | 100 | 1:0.5:1.5:2 | 0.038 | 6.39/39.23 | 8.00/41.52 |
| 54 | Beta-amylase | gi\|32400764 | *T. urartu* | 13 | 434 | 100 | 100 | 1:0.6:0.9:0.3 | 0.031 | 8.60/31.10 | 7.35/29.43 |
| 64 | Cytosolic 3-phosphoglycerate kinase | gi\|28172909 | *[Triticum aestivum](http://www.uniprot.org/taxonomy/4565)* | 2 | 195 | 100 | 100 | 1:2.6:0.9:0.7 | 0.027 | 4.91/31.26 | 5.42/31.10 |
| 40 | Triosephosphat-isomerase | gi\|11124572 | *Triticum aestivum* | 12 | 170 | 100 | 99.859 | 1:0.7:0.5:1.1 | 0.024 | 5.38/27.01 | 5.93/28.10 |
| 41 | Triosephosphat-isomerase | gi\|11124572 | *Triticum aestivum* | 13 | 326 | 100 | 100 | 1:0.7:1:1.7 | 0.018 | 5.38/27.01 | 6.42/27.01 |
| 2 | Predicted protein | gi\|326523589 | *[Hordeum vulgare subsp. spontaneum](http://www.uniprot.org/taxonomy/77009)* | 37 | 551 | 100 | 100 | 1:0.7:0.5:1.4 | 0.018 | 6.83/107.88 | 6.52/98.80 |
| 10 | Predicted protein | gi\|326493636 | *Hordeum vulgare subsp. vulgare* | 16 | 423 | 100 | 100 | 1:0.8:1.2:0.3 | 0.026 | 5.39/48.43 | 5.80/57.23 |
| 18 | Predicted protein | gi\|326494166 | *Hordeum vulgare subsp. vulgare* | 22 | 390 | 100 | 100 | 1:0.7:1:3.8 | 0.028 | 5.99/46.18 | 6.30/48.43 |
|  | **Proteometabolism** |  |  |  |  |  |  |  |  |  |  |
| 24 | Protein disulfide-isomerase precursor | gi\|1848212 | *Nicotiana tabacum* | 6 | 134 | 100 | 100 | 1:1.6:2.9:1.1 | 0.009 | 5.99/40.08 | 6.37/41.50 |
| 35 | Proteasome subunit alpha type 3 | gi\|50080306 | *Oryza sativa (japonica group)* | 13 | 353 | 100 | 100 | 1:0.6:0.7:4.2 | 0.041 | 5.76/27.49 | 6.07/29.80 |
| 42 | Proteasome | gi\|357159246 | *Arabidopsis thaliana* | 10 | 164 | 100 | 99.998 | 1:0.4:0.9:1.8 | 0.016 | 7.56/24.73 | 6.52/27.60 |
| 45 | Proteasome subunit beta type-4-like | gi\|357159415 | *Brachypodium distachyon* | 13 | 212 | 100 | 100 | 1:0.6:1.1:2.7 | 0.019 | 7.01/27.57 | 8.01/28.02 |
| 50 | Translocase of inner membrane 17 | gi\|301666340 | *Triticum aestivum* | 4 | 55 | 95.114 | 99.063 | 1:2.9:1.4:3.7 | 0.025 | 6.70/17.95 | 7.82/15.05 |
| 73 | Translocase of inner membrane 17 | gi\|301666340 | *[Triticum aestivum](http://www.uniprot.org/taxonomy/4565)* | 4 | 168 | 95 | 99 | 1:2.9:1.4:3.7 | 0.025 | 6.70/17.95 | 6.31/23.12 |
| 61 | Unnamed protein product | gi\|257307295 | *[Triticum aestivum](http://www.uniprot.org/taxonomy/4565)* | 20 | 531 | 100 | 100 | 1:0.4:0.8:0.5 | 0.018 | 5.14/71.39 | 5.01/68.32 |
| 28 | Predicted protein | gi\|326506152 | *Hordeum vulgare subsp. vulgare* | 9 | 137 | 100 | 99.994 | 1:2.2:0.9:1.2 | 0.037 | 5.15/31.52 | 5,56/40.45 |
| 44 | Predicted protein | gi\|326495678 | *Hordeum vulgare subsp. vulgare* | 14 | 257 | 100 | 100 | 1:0.9:1:1.9 | 0.018 | 6.33/27.55 | 6.65/28.40 |
|  | **Amino acid metabolism** |  |  |  |  |  |  |  |  |  |  |
| 6 | Methionine synthase | gi\|50897038 | *Hordeum vulgare subsp. vulgare* | 28 | 1230 | 100 | 100 | 1:0.8:0.7:2.9 | 0.032 | 5.68/84.79 | 6.48/84.86 |
| 7 | Methionine synthase 1 enzyme | gi\|68655495 | *Hordeum vulgare* | 9 | 319 | 100 | 100 | 1:0.7:0.7:1.9 | 0.016 | 5.74/84.85 | 6.20/84.90 |
| 12 | Serine hydroxymethyltransferase 1-like | gi\|357156812 | *Kordia algicida OT-1* | 17 | 498 | 100 | 100 | 1:0.6:0.7:2.3 | 0.031 | 6.58/51.81 | 7.38/64.05 |
| 43 | Glutathione transferase | gi\|20067415 | *Triticum aestivum* | 9 | 155 | 100 | 99.999 | 1:1.5:1.1:2.3 | 0.029 | 6.35/25.10 | 6.62/26.0 |
| 57 | Adenosylhomocysteinase | gi\|474160132 | *T. urartu* | 10 | 236 | 100 | 100 | 1:0.9:1.1:8.1 | 0.019 | 6.45/83.80 | 6.23/83.12 |
|  | **Nucleic acid metabolism** |  |  |  |  |  |  |  |  |  |  |
| 14 | UTP--glucose-1-phosphate | gi\|357156812 | *[Arabidopsis thaliana](http://www.uniprot.org/taxonomy/3702)* | 22 | 537 | 100 | 100 | 1:1.7:0.9:0.4 | 0.013 | 6.58/51.81 | 5.58/58.20 |
| 21 | Adenylosuccinate synthetase | gi\|6685803 | *Triticum aestivum* | 7 | 136 | 100 | 100 | 1:0.5:1.2:0.6 | 0.015 | 5.93/51.40 | 5.93/51.35 |
| 59 | Uridine 5'-monophosphate synthase | gi\|475599896 | *A. tauschii* | 13 | 183 | 100 | 100 | 1:1.3:2.6:3.9 | 0.023 | 7.10/45.51 | 7.09/44.23 |
| 62 | Adenylosuccinate synthetase | gi\|6685803 | *Triticum aestivum* | 7 | 136 | 100 | 100 | 1:0.4:0.9:0.9 | 0.025 | 5.93/51.40 | 6.12/50.13 |
|  | **Transcription and translation** |  |  |  |  |  |  |  |  |  |  |
| 33 | Elongation factor 1-beta | gi\|473753874 | *T. urartu* | 5 | 159 | 100 | 100 | 1:0.7:1.3:2.9 | 0.015 | 4.64/30.73 | 4.63/30.32 |
| 68 | Replication factor C like protein | gi\|27735373 | *Triticum aestivum* | 1 | 232 | 100 | 96 | 1:1.6:0.7:0.7 | 0.013 | 8.92/22.23 | 7.21/20.34 |
|  | **ATP synthesis** |  |  |  |  |  |  |  |  |  |  |
| 67 | ATP synthase beta subunit | gi\|525291 | *Triticum aestivum* | 19 | 544 | 100 | 100 | 1:1.9:1.5:2.9 | 0.018 | 5.56/59.33 | 4.98/58.23 |
|  | **Sucrose biosynthetic process** |  |  |  |  |  |  |  |  |  |  |
| 52 | Sucrose synthase type 2 | gi\|3393044 | *Triticum aestivum* | 31 | 636 | 100 | 100 | 1:0.7:0.7:2.3 | 0.031 | 6.17/93.06 | 5.21/93.12 |
| 53 | Phosphoglucomutase | gi\|18076790 | *Triticum aestivum* | 23 | 687 | 100 | 100 | 1:2.2:1.1:1.5 | 0.024 | 5.66/62.98 | 5.34/60.67 |
| 55 | Phosphoglucomutase | gi\|18076790 | *Triticum aestivum* | 18 | 476 | 100 | 100 | 1:0.5:1:1 | 0.029 | 5.66/62.98 | 5.23/59.61 |
|  | **Photosynthesis** |  |  |  |  |  |  |  |  |  |  |
| 74 | Ribulose-1,5-bisphosphate carboxylase/oxygenase large | gi\|521301434 | *T. urartu* | 11 | 131 | 100 | 100 | 1:0.9:0.9:2.2 | 0.018 | 6.04/53.34 | 6.43/54.56 |
| 8 | Predicted protein | gi\|326533328 | *Hordeum vulgare subsp. vulgare* | 29 | 769 | 100 | 100 | 1:0.5:2:0.9 | 0.015 | 5.36/61.72 | 5.38/64.0 |
|  | **Stress-related proteins** |  |  |  |  |  |  |  |  |  |  |
| 16 | Calreticulin-like protein | gi\|56606827 | *Triticum aestivum* | 15 | 194 | 100 | 100 | 1:0.5:0.7:2.5 | 0.014 | 4.49/47.40 | 4.49/47.40 |
| 13 | Calreticulin-like protein | gi\|56606827 | *Triticum aestivum* | 18 | 328 | 100 | 100 | 1:1.1:1:2.6 | 0.025 | 4.49/47.40 | 4.12/46.00 |
| 22 | Class II chitinase | gi\|62465514 | *Triticum aestivum* | 9 | 425 | 100 | 100 | 1:0.8:0.7:2.5 | 0.005 | 8.66/28.60 | 8.45/28.43 |
| 31 | pyridoxine biosynthesis protein | gi\|74026503 | *Triticum aestivum* | 11 | 88 | 99.851 | 0 | 1:1:1.2:0.5 | 0.017 | 6.4/33.51 | 6.5/32.41 |
| 47 | Manganese superoxide dismutase | gi\|1621627 | *Triticum aestivum* | 12 | 210 | 100 | 100 | 1:2.3:0.7:1 | 0.014 | 7.9/25.26 | 6.82/26.30 |
| 49 | Cold regulated protein | gi\|26017213 | *Triticum aestivum* | 9 | 140 | 100 | 99.505 | 1:1.5:0.8:0.6 | 0.024 | 4.84/17.79 | 4.48/20.50 |
| 51 | Cyclophilin | gi\|154761388 | *Triticum aestivum* | 9 | 240 | 100 | 100 | 1:2.4:7.1:29.1 | 0.023 | 8.59/18.62 | 9.0/17.0 |
| 65 | Pm3b-like disease resistance protein 15Q1 | gi\|134290443 | *T. aestivum* | 2 | 258 | 97 | 99 | 1:2.2:1.1:0.2 | 0.037 | 6.27/59.20 | 6.11/58.10 |
| 66 | Lactoylglutathione lyase | gi\|475531176 | *A. tauschii* | 12 | 405 | 100 | 100 | 1:1.9:0.9:0.9 | 0.014 | 5.43/32.83 | 5.23/32.67 |
| 69 | Glutathione S-transferase | gi\|5923877 | *T. aestivum* | 4 | 200 | 100 | 100 | 1:1:0.5:1.4 | 0.035 | 5.79/23.61 | 6.01/24.54 |
| 71 | Manganese superoxide dismutase | gi\|1621627 | *T. aestivum* | 7 | 80 | 100 | 99 | 1:2.3:0.7:1 | 0.015 | 7.90/25.26 | 7.12/20.32 |
| 72 | Peroxiredoxin-2E-1, chloroplastic | gi\|473825951 | *T. urartu* | 1 | 132 | 100 | 100 | 1:2:1:3.4 | 0.012 | 6.32/26.35 | 5.91/30.24 |
| 30 | Predicted protein | gi\|326493416 | *Hordeum vulgare subsp. vulgare* | 14 | 174 | 100 | 99.902 | 1:0.5:0.7:0.3 | 0.036 | 5.34/32.81 | 5.38/32.10 |
|  | **LEA protein** |  |  |  |  |  |  |  |  |  |  |
| 56 | Putative late embryogenesis abundant protein | gi\|154759403 | *Hordeum vulgare* | 14 | 293 | 100 | 100 | 1:1.1:0.8:0.4 | 0.026 | 6.95/52.19 | 9.31/80 |
| 15 | Em protein H5 | gi\|1169518 | *Triticum aestivum* | 4 | 174 | 100 | 100 | 1:0.8:0.7:0.2 | 0.035 | 5.14/10.01 | 5.56/20 |
|  | **Inhibitor** |  |  |  |  |  |  |  |  |  |  |
| 9 | Serpin-Z2B; AltName: Full=TriaeZ2b | gi\|75279909 | *Triticum aestivum* | 4 | 537 | 100 | 100 | 1:0.4:0.1:0.1 | 0.031 | 5.18/42.95 | 5.28/53.43 |
| 58 | Serpin-N3.2 | gi\|379060943 | *Triticum aestivum* | 15 | 867 | 100 | 100 | 1:2.2:0.9:1 | 0.036 | 5.18/43.03 | 5.21/40.12 |
| 60 | Serpin-N3.2 | gi\|379060943 | *Triticum aestivum* | 14 | 620 | 100 | 100 | 1:2.2:1:0.3 | 0.034 | 5.18/43.03 | 5.12/42.41 |
| 36 | Predicted protein | gi\|326489278 | *Hordeum vulgare subsp. vulgare* | 17 | 239 | 100 | 99.982 | 1:2.3:2.8:9 | 0.023 | 6.14/30.58 | 7.02/30.20 |
|  | **Storage proteins** |  |  |  |  |  |  |  |  |  |  |
| 63 | Globulin 3 | gi\|215398470 | *Triticum aestivum* | 20 | 489 | 100 | 100 | 1:0.8:0.7:2.5 | 0.006 | 7.78/66.65 | 7.16/59.61 |
|  | **Other proteins** |  |  |  |  |  |  |  |  |  |  |
| 3 | G356.108B02F010919 | gi\|32677110 | *Triticum aestivum* | 6 | 173 | 100 | 100 | 1:3.6:1.5:1.8 | 0.012 | 7.96/15.08 | 7.37/86.20 |
| 4 | G356.108B02F010919 | gi\|32677110 | *Triticum aestivum* | 8 | 523 | 100 | 100 | 1:2.2:1.1:1.5 | 0.023 | 7.96/15.08 | 7.48/84.20 |
| 17 | BJ291473 Y | gi\|23149830 | *Triticum aestivum* | 8 | 133 | 100 | 100 | 1:0.4:0.8:0.5 | 0.017 | 8.91/27.77 | 6.06/43.20 |
| 19 | Predicted protein | gi\|475536383 | *A. tauschii* | 9 | 295 | 100 | 100 | 1:3.1:2.3:2.2 | 0.026 | 5.89/43.41 | 5.89/48.24. |
| 20 | BJ297754 Y | gi\|23153402 | *Triticum aestivum* | 1 | 126 | 100 | 100 | 1:0.4:0.9:0.9 | 0.036 | 5.49/14.87 | 5.49/44.60 |
| 29 | Predicted protein | gi\|475585173 | *A. tauschii* | 20 | 687 | 100 | 100 | 1:0.6:1.3:1.6 | 0.031 | 7.26/27.94 | 8.00/38.52 |
| 32 | Predicted protein | gi\|326495978 | *Hordeum vulgare subsp. vulgare* | 6 | 134 | 100 | 99.984 | 1:1.8:1.8:2.8 | 0.017 | 4.21/12.68 | 3.63/30.50 |
| 34 | G550.120H01F010712 | gi\|32688377 | *Triticum aestivum* | 3 | 92 | 100 | 100 | 1:1.8:0.6:1.4 | 0.031 | 5.73/18.35 | 4.84/30.50 |
| 37 | G608.111J02F010910 | gi\|32766720 | *Triticum aestivum* | 7 | 122 | 100 | 98.307 | 1:2.2:2.2:0.6 | 0.009 | 8.68/16.53 | 7.60/30.30 |
| 38 | Predicted protein | gi\|326526175 | *Hordeum vulgare subsp. vulgare* | 9 | 166 | 100 | 100 | 1:1.5:0.7:1.6 | 0.015 | 6.19/26.04 | 4.50/26.80 |
| 39 | Os02g0634900 | gi\|115447473 | *Oryza sativa Japonica Group* | 12 | 286 | 100 | 100 | 1:0.2:0.9:1.6 | 0.027 | 5.39/29.83 | 5.39/29.83 |
| 46 | Predicted protein | gi\|475525322 | *A. tauschii* | 1 | 137 | 100 | 99 | 1:0.7:1.6:1.1 | 0.025 | 5.26/15.87 | 5.43/22.65 |
| 48 | Predicted protein | gi\|474071007 | *T. urartu* | 10 | 186 | 100 | 100 | 1:1.3:0.4:1.3 | 0.018 | 6.19/16.82 | 6.56/19.45 |
| 70 | Hypothetical protein TRIUR3_24891 | gi\|474407331 | *T. urartu* | 8 | 118 | 100 | 92 | 1:0.9:1:2.5 | 0.022 | 8.42/22.69 | 8.21/21.23 |
| **Endosperm** |  |  |  |  |  |  |  |  |  |  |  |
|  | **Storage proteins** |  |  |  |  |  |  |  |  |  |  |
| 8 | Gamma-gliadin | gi\|209971789 | *Aegilops tauschii* | 6 | 133 | 100 | 100 | 1:0.6:0.5:0.6 | 0.026 | 8.16/34.69 | 7.52/46.50 |
| 12 | Avenin-like protein | gi\|145321072 | *Triticum aestivum* | 12 | 101 | 100 | 100 | 1:1.3:1.2:2.6 | 0.032 | 8.29/33.70 | 7.38/42.50 |
| 14 | Alpha-gliadin | gi\|147883558 | *Triticum aestivum* | 1 | 73 | 99.955 | 100 | 1:0.8:1.2:6.7 | 0.039 | 7.62/35.53 | 6.69/39.50 |
| 20 | Alpha-gliadin | gi\|166406979 | *Triticum turgidum subsp.* | 1 | 55 | 95 | 99.995 | 1:2:1.1:1.9 | 0.036 | 8.58/33.09 | 7.22/35.50 |
| 22 | Alpha gliadin | gi\|154268814 | *Triticum aestivum* | 1 | 114 | 100 | 100 | 1:0.7:0.8:0.3 | 0.018 | 8.19/33.47 | 8.10/35.56 |
| 23 | Avenin-like protein | gi\|145321072 | *Triticum aestivum* | 3 | 60 | 98.607 | 99.969 | 1:1.4:0.7:0.4 | 0.013 | 8.29/33.70 | 6.88/34.80 |
| 24 | Alpha-gliadin | gi\|147883558 | *Triticum aestivum* | 1 | 132 | 100 | 100 | 1:0.2:0.1:0.3 | 0.028 | 7.62/35.53 | 7.55/35.83 |
| 25 | Gamma- gliadin | gi\|217039735 | *Triticum monococcum* | 2 | 119 | 100 | 100 | 1:0.7:0.9:0.5 | 0.016 | 8.32/22.11 | 7.67/37.80 |
| 26 | Avenin-like b5 | gi\|338817625 | *Triticum aestivum* | 6 | 84 | 99.56 | 97.771 | 1:0.7:0.9:3.2 | 0.013 | 8.82/33.76 | 7.58/38.50 |
| 27 | Avenin-like protein | gi\|156630232 | *Hordeum vulgare* | 5 | 151 | 100 | 100 | 1:1.1:0.8:0.4 | 0.023 | 7.83/33.37 | 7.68/34.60 |
| 28 | Avenin-like b1 | gi\|122232330 | *Triticum aestivum* | 6 | 106 | 99.997 | 99.947 | 1:1.3:1.3:2.1 | 0.035 | 8.08/33.79 | 7.90/38.60 |
| 33 | Gliadin/avenin-like seed protein | gi\|281335538 | *Triticum aestivum* | 4 | 86 | 99.723 | 99.356 | 1:0.6:0.7:0.4 | 0.023 | 6.2/22.81 | 6.58/24.80 |
| 34 | Gliadin/avenin-like seed protein | gi\|281335538 | *Triticum aestivum* | 2 | 227 | 100 | 100 | 1:0.5:0.4:0.3 | 0.033 | 6.2/22.81 | 7.02/21.65 |
|  | **Carbohydrate metabolism** |  |  |  |  |  |  |  |  |  |  |
| 4 | Beta amylase | gi\|32400764 | *Triticum aestivum* | 12 | 181 | 100 | 100 | 1:1:1.5:7.9 | 0.011 | 8.6/31.10 | 5.85/64.50 |
| 1 | Dihydrolipoyl dehydrogenase 1, mitochondrial | gi\|475592887 | *A. tauschii* | 19 | 257 | 95 | 100 | 1:1:0.4:0.9 | 0.017 | 7.63/58.81 | 6.53/80.05 |
| 5 | Dihydrolipoamide dehydrogenase precursor | gi\|13873336 | *Bruguiera gymnorrhiza* | 9 | 273 | 100 | 100 | 1:0.6:0.4:1.1 | 0.027 | 6.71/54.39 | 6.51/55.50 |
| 3 | Beta-D-glucan exohydrolase | gi\|20259685 | *Triticum aestivum* | 15 | 168 | 90 | 100 | 1:1.5:1.2:0.7 | 0.016 | 6.86/67.71 | 6.32/55.32 |
| 6 | beta-glucanase | gi\|600857 | *Arabidopsis thaliana* | 4 | 150 | 100 | 100 | 1:3.3:4.3:2.2 |  | 8.71/35.50 | 8.29/53.80 |
| 29 | Dihydrolipoyl dehydrogenase 1, mitochondrial | gi\|475592887 | *A. tauschii* | 23 | 795 | 100 | 100 | 1:1:2.6:0.8 | 0.018 | 7.63/58.81 | 8.00/38.52 |
| 11 | Glyceraldehyde-3-phosphate dehydrogenase | gi\|120680 | *Hordeum vulgare* | 11 | 345 | 100 | 100 | 1:0.6:0.4:0.7 | 0.029 | 6.67/36.60 | 6.72/45.80 |
| 13 | Cytoplasmic aldolase | gi\|218157 | *Oryza sativa Japonica Group* | 7 | 76 | 99.504 | 96.105 | 1:0.7:0.7:1.5 | 0.014 | 6.56/39.15 | 7.51/42.50 |
|  | **Proteometabolism** |  |  |  |  |  |  |  |  |  |  |
| 30 | Proteasome subunit alpha type-3 | gi\|474156765 | *T. urartu* | 21 | 632 | 100 | 100 | 1:0.6:1:1.2 | 0.018 | 5.82/27.42 | 5.83/28.64 |
|  | **Amino acid metabolism** |  |  |  |  |  |  |  |  |  |  |
| 2 | Methionine synthase | gi\|6006863 | *A. thaliana* | 1 | 157 | 99 | 99 | 1:2:2.6:2.6 | 0.025 | 6.09/84.15 | 6.32/78.56 |
|  | **Inhibitor** |  |  |  |  |  |  |  |  |  |  |
| 7 | Serpin-Z2B | gi\|75279909 | *Triticum aestivum* | 14 | 1000 | 100 | 100 | 1:1.1:2:1.7 | 0.027 | 5.18/43.01 | 5.48/46.80 |
| 10 | Serpin-Z2A | gi\|75313847 | *Triticum aestivum* | 11 | 738 | 100 | 100 | 1:1.7:4.9:2.9 | 0.018 | 5.46/43.34 | 5.63/44.60 |
| 32 | Xylanase inhibitor protein I | gi\|20804336 | *Triticum aestivum* | 14 | 570 | 100 | 100 | 1:0.9:2.3:3.5 | 0.015 | 8.66/33.48 | 8.49/32.50 |
|  | **Stress-related proteins** |  |  |  |  |  |  |  |  |  |  |
| 9 | HSP70 | gi\|2827002 | *Triticum aestivum* | 14 | 407 | 100 | 100 | 1:1:2.1:0.9 | 0.028 | 5.14/71.39 | 6.42/44.50 |
| 21 | Peroxidase 1 | gi\|22001285 | *Triticum aestivum* | 12 | 288 | 100 | 100 | 1:0.2:1:0.8 | 0.014 | 8.14/39.26 | 8.32/39.80 |
| 16 | Class II chitinase | gi\|62465514 | *Triticum aestivum* | 9 | 425 | 100 | 100 | 1:0.5:0.4:0.8 | 0.013 | 8.66/28.60 | 7.01/33.20 |
| 17 | Lactoylglutathione lyase | gi\|475531176 | *A. tauschii* | 10 | 129 | 90 | 100 | 1:1.1:1:0.5 | 0.033 | 5.43/32.83 | 7.90/37.80 |
| 18 | Translationally controlled tumor protein | gi\|21070379 | *Triticum aestivum* | 3 | 264 | 100 | 100 | 1:1.2:2.4:3.2 | 0.028 | 4.55/18.85 | 4.34/40.56 |
| 19 | 1-Cys peroxiredoxin PER1 | gi\|475442339 | *A. tauschii* | 18 | 565 | 100 | 100 | 1:1.4:1.2:3.6 | 0.025 | 6.31/24.19 | 8.12/32.24 |
| 31 | 27K protein | gi\|30793446 | *Triticum aestivum* | 10 | 203 | 100 | 100 | 1:0.9:1.2:0.6 | 0.018 | 6.06/23.27 | 6.39/25.60 |
|  | **Other proteins** |  |  |  |  |  |  |  |  |  |  |
| 15 | Predicted protein | gi\|326497973 | *Hordeum vulgare subsp. vulgare* | 1 | 212 | 100 | 100 | 1:2.1:2.2:7.2 | 0.012 | 6.27/39.06 | 6.81/37.50 |

a) Pep. Count: matched peptide count.

b) Protein Score: statistical probability of true positive identification of the predicted protein calculated by MASCOT with 0.3 peptide tolerance and one allowed missed cleavage (score ≥42 against NCBInr, ≥66 against MSDB, or ≥55 against Swiss-Prot).

c) *TpI*/TMW (kDa): p*I* of predicted protein/molecular mass of predicted protein.

d) *EpI*/EMW (kDa): p*I* of protein on the gel/molecular mass of protein on the gel.

**Table S2:** The unnamed and predicted proteins from embryo and endosperm in Table S1 grouped by BLASTP (www.ncbi.nlm.nih.gov/BLAST/) in searching for closest homologue.

| **Spot No.** | **Accession**  **no. ^a^** |  | **Homologue** |  |  |  |  |
| --- | --- | --- | --- | --- | --- | --- | --- |
|  |  | **NCBI accession no. ^b^** | **Protein Name** | **Species** | **Id% ^c^** | **Ps% ^d^** | **QC%^e^** |
| **Embryo** |  |  |  |  |  |  |  |
| **Carbohydrate metabolism** |  |  |  |  |  |  |  |
| 2 | gi\|326523589 | XP_003558862 | Putative aconitate hydratase, cytoplasmic | *Brachypodium distachyon* | 93% | 95% | 99% |
| 10 | gi\|326493636 | [AGH20061.1](http://www.ncbi.nlm.nih.gov/protein/461744056?report=genbank&log$=prottop&blast_rank=3&RID=87DA4CNK014) | Enolase | *Triticum aestivum* | 99% | 99% | 100% |
| 18 | gi\|326494166 | [EMT15199.1](http://www.ncbi.nlm.nih.gov/protein/475567132?report=genbank&log$=prottop&blast_rank=3&RID=87E48268014) | Putative Isocitrate dehydrogenase (NADP), chloroplastic | *Aegilops tauschii* | 99% | 100% | 100% |
| **Proteometabolism** |  |  |  |  |  |  |  |
| 61 | gi\|257307295 | [AAB99745.1](http://www.ncbi.nlm.nih.gov/protein/2827002?report=genbank&log$=prottop&blast_rank=1&RID=87EVSZTU014) | HSP70 | *Triticum aestivum* | 100% | 100% | 100% |
| 28 | gi\|326506152 | [EMT13225.1](http://www.ncbi.nlm.nih.gov/protein/475558943?report=genbank&log$=prottop&blast_rank=2&RID=87EXWD80014) | Proteasome subunit alpha type-1 | *Aegilops tauschii* | 98% | 98% | 91% |
| 44 | gi\|326495678 | [EMT09268.1](http://www.ncbi.nlm.nih.gov/protein/475538570?report=genbank&log$=prottop&blast_rank=2&RID=87F5902W015) | Proteasome subunit alpha type-6 | *Aegilops tauschii* | 99% | 100% | 100% |
| **Photosynthesis** |  |  |  |  |  |  |  |
| 8 | gi\|326533328 | [XP_003558045.1](http://www.ncbi.nlm.nih.gov/protein/357112497?report=genbank&log$=prottop&blast_rank=2&RID=87FAXMAF015) | RuBisCO large subunit-binding protein subunit alpha | *Brachypodium distachyon* | 94% | 96% | 99% |
| **Stress-related proteins** |  |  |  |  |  |  |  |
| 30 | gi\|326493416 | [EMT08036.1](http://www.ncbi.nlm.nih.gov/protein/475531176?report=genbank&log$=prottop&blast_rank=2&RID=87FE31FM015) | Lactoylglutathione lyase | *Aegilops tauschii* | 98% | 100% | 100% |
| **Inhibitor** |  |  |  |  |  |  |  |
| 36 | gi\|326489278 | [XP_003579914.1](http://www.ncbi.nlm.nih.gov/protein/357163990?report=genbank&log$=prottop&blast_rank=2&RID=87FHY56M014) | Prohibitin-3, mitochondrial-like | *Brachypodium distachyon* | 96% | 96% | 100% |

a Accession number of the predicted protein in Table S1.

b Accession number of the homologue.

c Identity %.

d Positives %.

e Query coverage %.
