# Supplementary material for: Comparative proteome analysis of embryo and endosperm reveals central differential expression proteins involved in wheat seed germination
Source: BMC Plant Biol. 2015 Apr 8;15:97. doi: 10.1186/s12870-015-0471-z (PMC4407426; doi:10.1186/s12870-015-0471-z)
Supplement: Additional file 4: Table S4. — Functions and expression features of some important DEPs in wheat embryo and endosperm. [file 12870_2015_471_MOESM4_ESM.docx]

**Additional file 4: Table S4** **Functions and expression features of some important DEPs in wheat embryo and endosperm**

| **Functions** | **Protein name** | **Accession No**. | **Species** | **Embryo expression trend** | **Endosperm expression trend** |
| --- | --- | --- | --- | --- | --- |
| **Carbohydrate metabolism** | Alcohol dehydrogenase ADH1A | gi\|119388731 | *Triticum turgidum subsp. dicoccon* | M23 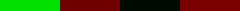 | + |
|  | Glyceraldehyde-3-phosphate dehydrogenase | gi\|253783729 | *Triticum aestivum* | M26 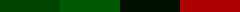 | N11 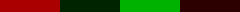 |
|  | Beta-amylase | gi\|32400764 | *Triticum. urartu* | M54 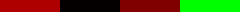 | N4 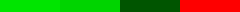 |
|  | Fructose-bisphosphate aldolase | gi\|226316439 | *Secale cereale* | M25 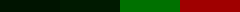 | + |
|  | Dihydrolipoyl dehydrogenase 1 | gi\|357134512 | *Arabidopsis thaliana* | M11 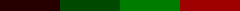 | N1 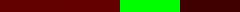 |
| **Proteometabolism** | Protein disulfide-isomerase precursor | gi\|1848212 | *Nicotiana tabacum* | M24 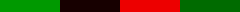 | + |
|  | Translocase of inner membrane 17 | gi\|301666340 | *Triticum aestivum* | M50 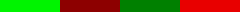 | + |
|  | Proteasome subunit alpha type-3 | gi\|474156765 | *Triticum urartu* | M35 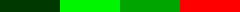 | N30 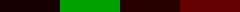 |
| **Amino acid metabolism** | Methionine synthase | gi\|50897038 | *Hordeum vulgare subsp. vulgare* | M6 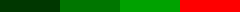 | N2 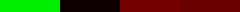 |
| **Nucleic acid metabolism** | UTP--glucose-1-phosphate | gi\|357156812 | *Arabidopsis thaliana* | M14 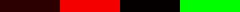 | + |
| **Stress**-**related proteins** | Calreticulin-like protein | gi\|56606827 | *Triticum aestivum* | M16 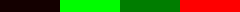 | + |
|  | Class II chitinase | gi\|62465514 | *Triticum aestivum* | M22 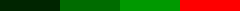 | N16 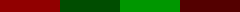 |
|  | HSP70 | gi\|2827002 | *Triticum aestivum* | - | N9 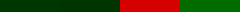 |
|  | Lactoylglutathione lyase | gi\|475531176 | *Aegilops tauschii* | M66 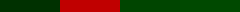 | N17 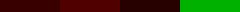 |
|  | Cyclophilin | gi\|154761388 | *Triticum aestivum* | M51 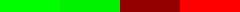 | + |
|  | Manganese superoxide dismutase | gi\|1621627 | *Triticum aestivum* | M47 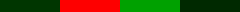 | + |
|  | Glutathione S-transferase | gi\|5923877 | *Triticum aestivum* | M69 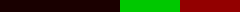 | + |
| **Photosynthesis** | Ribulose-1,5-bisphosphate carboxylase/oxygenase large | gi\|521301434 | *Triticum urartu* | M74 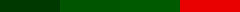 | - |
| **ATP synthesis** | ATP synthase beta subunit | gi\|525291 | *Triticum aestivum* | M67 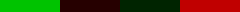 | + |
| **Sucrose synthesis** | Sucrose synthase type 2 | gi\|3393044 | *Triticum aestivum* | M52 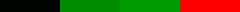 | + |
| **Storage proteins** | Gamma-gliadin | gi\|217039735 | *Triticum monococcum* | + | N25 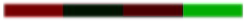 |
|  | Alpha gliadin | gi\|154268814 | *Triticum aestivum* | + | N22 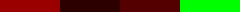 |
|  | Avenin-like protein | gi\|156630232 | *Hordeum vulgare* | - | N27 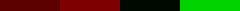 |
| **Inhibitor** | Xylanase inhibitor protein I | gi\|20804336 | *Triticum aestivum* | + | N32 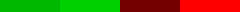 |
|  | Serpin-N3.2 | gi\|379060943 | *Triticum aestivum* | M58 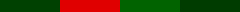 | + |
|  | Serpin-Z2B | gi\|75279909 | *Triticum aestivum* | M9 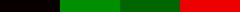 | N7 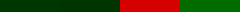 |

“+” indicates the protein expression without 2-fold differences. “-” indicates the proteins that are not detectable.
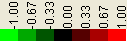
.
