# Supplementary material for: Comparative proteome analysis of embryo and endosperm reveals central differential expression proteins involved in wheat seed germination
Source: BMC Plant Biol. 2015 Apr 8;15:97. doi: 10.1186/s12870-015-0471-z (PMC4407426; doi:10.1186/s12870-015-0471-z)
Supplement: Additional file 6: Table S6. — The specific primer sequences for qRT-PCR assays. [file 12870_2015_471_MOESM6_ESM.docx]

**Additional file 6: Table S6.** The specific primer sequences for the qRT-PCR assays.

| **Gene Name** | **Forward primer** | **Reverse primer** |
| --- | --- | --- |
| **embyro** |  |  |
| alcohol dehydrogenase ADH1A gene | CGGAATTTGTGAACCCGAAA | GCGGTCAACTCCACCATTTG |
| glyceraldehyde-3-phosphate dehydrogenase | TCAAATGCTAGCTGCACCAC | CACCTCTCCAGTCCTTGCTC |
| methionine synthase gene | TAAGTCATGGCTCGCGTTTG | CATCCTTTTGACCAGCCAATG |
| **endosperm** |  |  |
| β-glucanase gene | GGAACACCGCGATCTTGCT | ATGGGCTGGAGGCAGATTTT |
| heat shock protein 70(HSP70) gene | TCTCCTCCATGGTGCTTATCAAG | GGTGACCACGGCGTTCTT |
| xylanase inhibitor protein I gene | GGTACACCCCAAGAACGTCTACTAC | ATCGGTCCCAGAGCATGATG |
| **Reference gene** |  |  |
| adenosine diphosphate | GCTCTCCAACAACATTGCCAAC | GCTTCTGCCTGTCACATACGC |
